# A scoping review of the ethics frameworks describing issues related to the use of extended reality

**DOI:** 10.12688/openreseurope.17283.2

**Published:** 2025-02-10

**Authors:** Shereen Cox, Alina Kadlubsky, Ellen Svarverud, Jonathan Adams, Rigmor C. Baraas, Rosemarie D.L.C Bernabe

**Affiliations:** 1 Universitetet i Oslo, Oslo, Oslo, Norway; 2 Open AR Cloud Europe gUG, Munich, Germany; 3 University of South-Eastern Norway, Kongsberg, Buskerud, Norway

**Keywords:** Virtual reality, augmented reality, mixed reality, ethics, extended reality, immersive technologies

## Abstract

The use of extended reality (XR) / immersive technologies such as virtual, augmented, mixed reality and virtual worlds (Metaverse) raises issues of ethical concern. The various issues, if left unaddressed, may impact human wellbeing over time. Immersive technologies are used in entertainment, commerce, training, education, health, and the military among others. Subsequently, there is a broad spectrum of users with various degrees of competencies and vulnerabilities. Special attention regarding long-term effects of immersive technologies on children and the lack of consideration of inclusivity for all persons in society is essential. Several publications have highlighted ethical issues related to immersive technologies, and some have sought to address these issues by proposing solutions or approaches in the form of frameworks, codes of conduct or best practices. This review examined literature between 2000 and 2023 to identify proposed or adopted ethical frameworks, codes of conduct or best practices for immersive technologies. Qualitative research method was applied, using a scoping review approach. Twenty-eight papers were selected for analysis. Approximately 70% of the selected papers were published between 2020 and 2022. Using an inductive thematic analysis method, seven fundamental values and twenty-two corresponding principles were generated. The main values are respect for persons, well-being, safety, integrity and trust, justice, and responsiveness. The dominant principles identified are privacy, informed consent, responsibility, transparency, and freedom. The authors of the papers were predominantly academic researchers. The normative approaches to addressing ethical issues were organised into four domains: society and governance, industry, research/academic organisations, and individuals. Recommendations are: 1) development and/or application of laws or guidelines to ethical, legal, and social issues with immersive technologies; 2) adoption of inclusive approaches to design and development; 3) minimisation of risk for research participants; 4) empowerment of users of immersive technologies; and 5) promotion of responsibility and sincerity in the use of virtual space, especially in matters concerning identity and conduct.

## Plain language summary

The use of technologies like virtual reality, augmented reality, mixed reality, and the Metaverse raises ethical concerns. If these concerns are not addressed, they may negatively affect people's well-being. Immersive technologies are used in various areas such as entertainment, business, education, healthcare, and the military. This means there are different types of users with different levels of skills and vulnerabilities. We need to pay special attention to the long-term effects of these technologies and ensure that inclusivity is considered for everyone in society. Numerous publications have identified ethical issues related to immersive technologies and have proposed solutions in the form of frameworks, codes of conduct, and best practices mitigating risk.

To explore the different ethical approaches, we conducted a review of relevant literature from 2000 to 2023. Our analysis focused on 28 papers, with around 70% of them published between 2020 and 2022. Through this analysis, we identified seven core values and twenty-two corresponding principles. The key values include respect for individuals, well-being, safety, integrity and trust, justice, and responsiveness. The most frequently mentioned principles include privacy, informed consent, responsibility, transparency, and freedom. Academic researchers authored many of the papers. The ethical approaches were categorized into the following four domains: 1) society and governance, 2) industry, 3) research/academic organizations, and 4) individuals. Based on our findings, we recommend the following: 1) laws and/or guidelines should be developed and/or applied to ethical, legal, and social issues with immersive technologies; 2) industry practitioners should adopt inclusive approaches to design and development; 3) developers and researchers should minimise risk for research participants; 4) empowerment of users of immersive technologies; and 5) promotion of responsibility and sincerity in the use of virtual space, specially in matters concerning identity and conduct.

## Introduction

Extended reality (XR) technologies, also termed immersive technologies, are emerging technologies characterised by “immersive video content, enhanced media experiences, and interactive and multi-dimensional human experiences” ( Pearlman, 2020). XR is an umbrella term referring to virtual (VR), augmented (AR) and mixed reality (MR). Since the turn of the millennium, our technological landscape has been marked by ever-increasing “convergence”, a word commonly used to describe the phenomenon by which early smartphones began to provide features like cameras, calendars, basic internet access, and even simple games, besides making phone calls ( Adams *et al.*, 2018). Two decades later, XR can be viewed as the next level of convergence, where not only different technical capabilities converge on a device (and between devices and networks) but where the digital world will itself converge with the physical world for the very first time. However, XR and its related technologies are double-edged swords. Their usefulness and potential are undeniable: not only are they quickly moving towards ubiquity; their fields of application are virtually endless. At the same time, for these technologies to function, users and their environments may be exposed to new risks and other ethical/philosophical issues that may have negative and/or unintended effects and/or consequences. As the technology is developing and becoming more common, Europe must strive to establish an ethical governance/policy framework for XR technologies that preserve European values and virtues while guiding not only users but also, most significantly, designers and developers of the technology ( Fox & Thornton, 2022; Stephens, 2022). This is the first of a series of articles that explore ethical issues related to the development and use of XR and its related technologies. To begin the work of providing a normative foundation for ethical reflection on the development and use of immersive technologies, this article explores the ethical principles and related ethical issues in XR development and use from proposed ethical frameworks, codes of conduct, and good practices in published literature.

### Research question

What are the foundational ethical principles governing XR and the relevant ethical issues according to extant ethical frameworks, codes of conduct, and good practices?

### Specific aim and objectives

**Aim:** To provide an overview of ethical principles and relevant ethical issues according to ethical frameworks, codes of conduct and good practices related to XR.

**Specific objectives:**

- To assess foundational ethical principles in the noted guidelines.
- To identify ethical issues addressed and the means proposed to address the identified issues and note the commonalities and/or differences in the various approaches.
- To identify gaps in ethical issues addressed in the noted guidelines.

## Methods

This paper adopted the PRISMA-ScR (Preferred Reporting Items for Systematic Reviews and Meta-Analyses extension for Scoping Reviews) research methodology, an approach meant to map relevant literature on a research subject/area ( Arksey & O'Malley, 2005; Moher *et al.*, 2009). A preliminary literature search did not reveal any other study mapping the ethics guidelines for immersive technologies. However, Jobin *et al.* (2019) performed a global landscape of ethics guidelines for artificial intelligence. They developed a protocol based on the PRISMA framework which was pilot-tested and calibrated. Given the similarity of the research question, we adopted the protocol outlined by Jobin et al. for this scoping review within the context of ethics guidelines for immersive technologies. We note that their focus was on grey literature which guided us to expand our search to include both grey and academic literature. The search documentation can be found in Zenodo ( Cox *et al.*, 2024).

### Protocol and registration

The authors did not prepare a protocol.

### Data sources and search strategy

The research team for this scoping review was multi-disciplinary, comprising academic researchers and practitioners with expertise in ethics, philosophy, neuroscience, and industry practitioners working with immersive technologies. In January 2023, the research team developed and agreed on the search terms in accordance with a priori knowledge and experience with immersive technologies. The databases, Scopus and the Institute of Electrical and Electronics Engineers (IEEE), were identified as suitable for a comprehensive search (see Table 1). The approved search terms were submitted to the medical librarian at the University of Oslo, Faculty of Medicine, who completed the database search on February 8, 2023. The date limit for the search was 2000–2023.

**Table 1.** Search terms used by the librarian and number of abstracts retrieved for analysis.

Database	Search terms	Number of retrieved abstracts	
**Scopus**	( TITLE-ABS-KEY ( ethic* OR bioethic* OR philosoph* OR integrity OR moral* OR "human centred" OR "human lefted" ) AND TITLE-ABS-KEY ( framework* OR guideline* OR guidance* OR regulation* OR regulatory OR law OR laws OR legislat* OR legal OR norm OR norms OR normative OR "code of conduct" OR ( good W/2 practice* ) ) AND TITLE-ABS-KEY ( ( interactive W/2 ( media* OR experience* ) ) OR metaverse OR xr OR xr4* OR ( immersive W/2 ( technolog* OR environment* ) ) OR ( virtual W/2 ( realit* OR environment* ) ) OR ( ( virtualis* OR virtualiz* ) W/2 ( technolog* OR network* ) ) OR ( augmented W/2 realit* ) OR ( mixed W/2 realit* ) OR ( extended W/2 realit* ) ) )	**1236**	
**IEEE**	(( ( interactive NEAR/2 ( media OR experience* ) ) OR metaverse OR xr OR xr4* OR ( ** immersive NEAR/2 (technology OR technologies)) OR (immersive NEAR/2 (environment** OR environments)) OR ( virtual NEAR/2 ( reality OR realities OR environment* ) ) OR ( virtuali* NEAR/2 ( technolog* OR network* ) ) OR ( augmented NEAR/2 (reality OR realities)) OR ** ( mixed NEAR/2 (reality OR realities) ) OR ( extended NEAR/2 (reality OR** ** realities))) AND ( framework OR frameworks OR guideline OR guidelines OR guidance OR ** **guidances OR regulation OR regulations OR regulatory OR law OR laws OR legislation** ** OR legislations OR legislative OR legal OR norm OR norms OR normative OR "code of** conduct" OR ( good NEAR/2 practice*)) AND ( ethic OR ethical OR ethics OR ethically OR bioethic OR bioethical OR philosophi* OR integrity OR integrities OR moral OR morals **OR morality OR moralities OR "human centred" OR "human lefted" ))**	**352**	
**Summary**		**Total number of** ** records**	**1588**
		**After** ** deduplication**	**1359**

Following the methodology outlined by Jobin *et al.* (2019), two researchers (SC and JA) each performed a Google search while logged out of personal accounts, in private browsing mode and with web history, cache, and cookies deleted. Every link in the first 100 results was followed and screened for frameworks or guidelines, which identified further documents for inclusion. The search identified academic peer-reviewed articles and grey literature from academic, governmental, or non-governmental organisations and conferences. One important search finding was a news article highlighting the South Korean Government's publication of ethics guidelines for the Metaverse ( Ministry of Science and ICT, 2022). SC conducted an exhaustive citation chaining for selected full-text documents, but only three met the inclusion criteria. The screening and full-text reading were conducted between February 10 and March 17, 2023. Although approximately 22 documents were identified during citation chaining, only three were included. Since a recently published document was identified as relevant based on the eligibility criteria, it was manually inputted as it was published after the initial database search.

### Title and abstract relevance screening

The citations were uploaded into the web-based platform Rayyan for screening and selection of abstracts based on eligibility criteria outlined in Table 2 ( Ouzzani *et al.*, 2016). Initial screening was done by SC, and a secondary screening by ES. RdcB reviewed to decide on unresolved conflicts. Documents were excluded if there was no reference to any of the various forms of immersive technologies, i.e., virtual reality, augmented reality, mixed reality, or the Metaverse. Further exclusion was based on whether the documents did not discuss nor utilise a normative approach to ethical issues with immersive technologies or if the authors discussed immersive technologies without reference to ethics. Additionally, documents where full texts could not be retrieved were excluded.

**Table 2.** Eligibility criteria. The organisation of this table is adapted from: Jobin, A., Ienca, M. & Vayena, E. The global landscape of AI ethics guidelines. Nat Mach Intell 1, 389–399 (2019). https://doi.org/10.1038/s42256-019-0088-2 then adjusted based on the search criteria related to ethics guidelines for immersive technologies.

Screening	
**Sources** ** considered**	**Types:** Abstract and citation databases of scholarly (academic) literature, guidelines, recommendations, policy documents, regulations, framework, official standards
	**Issuers:** Academics, institutions, government and affiliated agencies, non-governmental organisations, professional societies, non-profit organisations
	**Language**: English only
**Sources excluded**	Conference summaries, papers reporting the results of interventional studies using immersive technologies, videos, images, podcasts, blog articles, journalistic articles, syllabi, books
Eligibility	
**Sources** ** included**	Explicitly refer to "immersive technologies" or extended reality or virtual reality or mixed reality, or augmented reality, or the Metaverse
	Self-proclaim in the title or abstract to be a guideline, code of ethics, code of conduct, best practice, or ethics framework for virtual reality, mixed reality, augmented reality or Metaverse
	Expressed in normative or prescriptive language (modal verbs or imperatives)
	Generally guiding (i.e., covers all or several fields/applications)
**Sources** ** excluded**	Do not mention immersive technologies, extended reality, virtual reality, or the Metaverse.
	Do not identify itself as an ethics guideline, code, code of conduct, best practice, or framework
	Not expressed in normative or prescriptive language
	Narrowly field-specific

### Data charting process - synthesis and coding strategy

Twenty-eight full-text documents were identified for coding and data analysis. Documents were included if the authors indicated in the title and/or abstract that they were proposing/prescribing a code of ethics, a guideline or guidance, best practice, or a framework for any or all immersive technologies. All documents were in English based on the search criteria. After the initial title and abstract screening, we excluded 1344 documents that did not meet our eligibility criteria. Fifteen documents were selected from abstract and title screening. We identified additional 30 documents through google searches, citation chaining and manual input. In total, there were 45 full text documents for screening; 17 texts were excluded for not meeting the eligibility criteria outlined above. Subsequently, 28 documents were uploaded to NVIVO version 1.7.1 for coding ( QSR International, 2018). Although the NVIVO software was used for coding, due to licensing requirements, the codes were extracted from NVIVO and organised in Microsoft excel spreadsheet to enable open access and uploaded to Zenodo ( Cox *et al.*, 2024). The files were copied and shared among five researchers (SC, ES, AK, JA, and RdcB) who individually coded based on the pre-determined categories: 1) Ethical issues, 2) Foundational ethical principles and 3) Approaches to addressing the identified ethical issues. Although the main thematic categories were pre-determined, the coding of the texts was entirely inductive.

**Figure 1.**
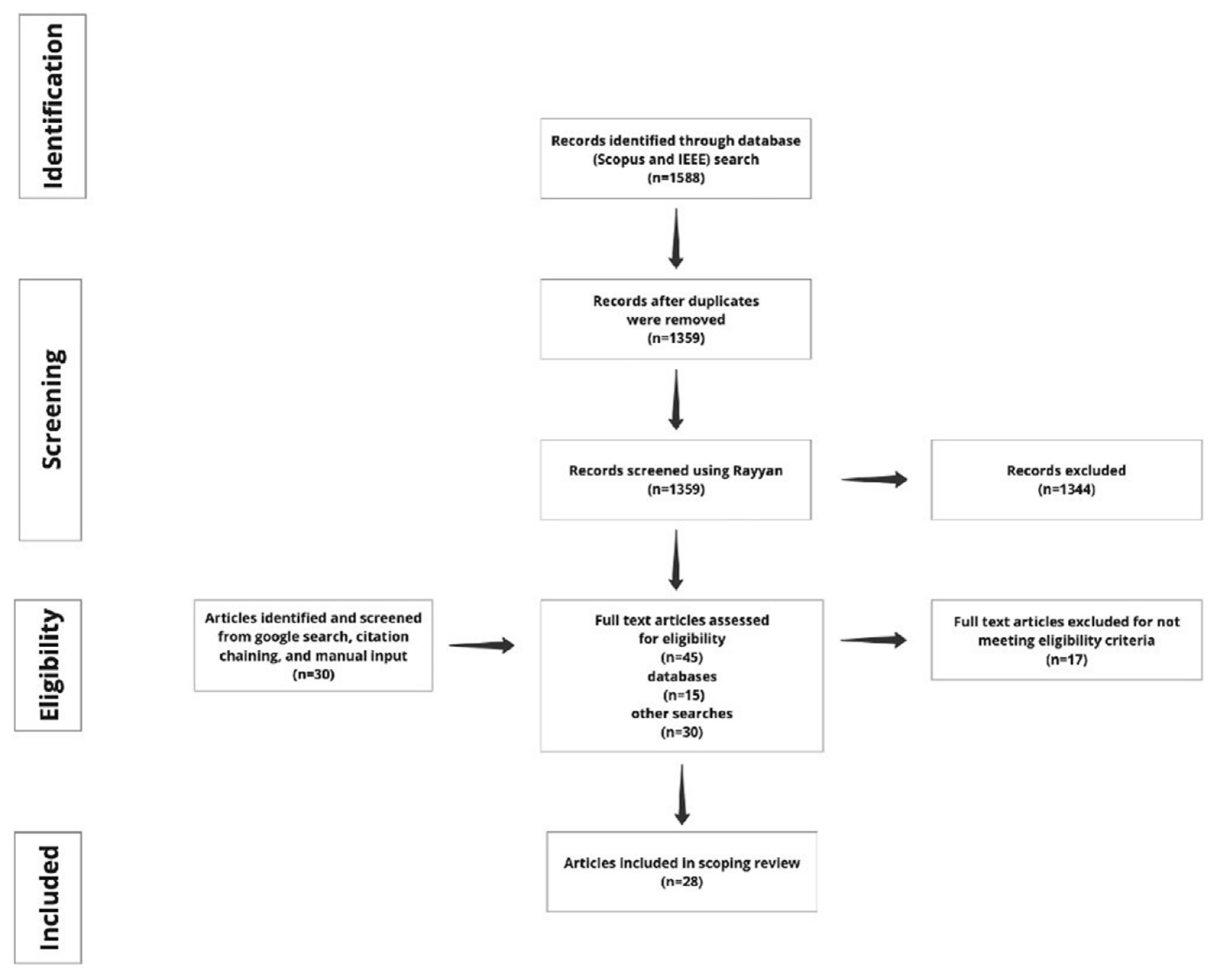
PRISMA flow diagram explaining the retrieval and screening process. The search sources for the databases: Scopus and IEEE, Google search, citation chaining and manual input ( Moher *et al.*, 2009).

To minimize potential bias due to interpretation, the coded ethical issues and foundational principles closely reflect the original text in the reviewed publications. However, the value categories were derived through collaborative discussions and the shared understanding of the principles by the researchers. The researchers met weekly to discuss the identified codes and explanations given for each. Codes similar in meaning or context were merged. However, a consensus was not sought for the inclusion of dissimilar codes. The reason was to ensure that the findings were reflective of the variations of focus in relation to the author, target audience or topic of discussion. Figure 1 is a schematic representation of the retrieval and screening process. Table 3 outlines the 28 documents, the type of immersive technology discussed, the target audience and the geographic location of the first author or organisation.

**Table 3.** Summary of documents by type of technology, country of the author, and target audience.

Year of publication	Title	Type or XR	Type of issuers	Country of issuers	Target audience	Method of retrieval
**2000**	New Directions: A Value-Sensitive Design Approach to Augmented Reality ( Friedman & Kahn, 2000)	AR	Academic	USA	Developers or designers	Citation chaining
**2005**	Some Practical Considerations of Ethical Issues in VR Research ( Behr *et al.*, 2005)	VR	Academic	Netherlands	Researchers	Other search
**2008**	Meta-Ethics for the Metaverse: The Ethics of Virtual Worlds ( Spence, 2008)	Metaverse	Academic	Australia	Designers, administrators, and players	Scopus/IEEE
**2016**	Real Virtuality: A Code of Ethical Conduct. Recommendations for Good Scientific Practice and the Consumers of VR-Technology ( Madary & Metzinger, 2016)	VR	Academic	Germany	Researchers	Scopus/IEEE
**2017**	Asking Ethical Questions in Research using Immersive Virtual and Augmented Reality Technologies with Children and Youth ( Southgate *et al.*, 2017)	XR	Academic	Australia	Researchers	Scopus/IEEE
	Ethical Considerations for the Use of Virtual Reality: An Evaluation of Practices in Academia and Industry ( Luro *et al.*, 2017)	VR	Academic	Sweden	Academic and industrial practitioners	Scopus/IEEE
**2019**	Ethically Aligned Design: A Vision for Prioritising Human Well-being with Autonomous and Intelligent Systems ( The IEEE Global Initiative on Ethics of Autonomous and Intelligent Systems, 2019)	XR	Non-profit organisation (NPO)	USA	Academic and industrial practitioners	Other search
	Ethical Guidelines in Virtual Reality: Towards a Code of Conduct in Research ( Bliznyuk, 2019)	VR	Academic	Germany	Unspecified	Other search
**2020**	Immersive Technology Standards for accessibility, inclusion, ethics, and safety ( Boyd-Noronha, 2020)	XR	NPO	USA	Public-private industries, governments, and academic organisations	Other search
	The Ethics of Realism in Virtual and Augmented Reality ( Slater *et al.*, 2020)	VR, AR	Academic	UK	Researchers, content creators, and distributors of XR systems	Citation chaining
	A Self-Guiding Tool to Conduct Research With Embodiment Technologies Responsibly ( Aymerich-Franch & Fosch-Villaronga, 2020)	VR, Avatars	Academic	Netherlands	Researchers and developers	Scopus/IEEE
	The XRSI Privacy Framework ( Pearlman, 2020)	XR	NPO	International	Public-private industries, governments and academic organisations.	Other search
**2021**	ARLEAN: An Augmented Reality Learning Analytics Ethical Framework ( Christopoulos *et al.*, 2021)	AR	Academic	Finland, Greece	Designers and educators	Scopus/IEEE
	E3XR: An Analytical Framework for Ethical, Educational and Eudaimonic XR Design ( Lee & Hu-Au, 2021)	XR	Academic	USA	Designers and educators	Scopus/IEEE
	The IEEE Global Initiative on Ethics of Extended reality (XR) Report: Social and multi-user spaces in VR, trolling, Harassment, and online safety ( Cortese & Outlaw, 2021)	XR	NPO	USA	public-private industries, governments, and academic organisations	Other search
	The IEEE Global Initiative on Ethics of Extended reality (XR) Report: Who owns our second lives: Virtual clones and the right to your identity ( Eriksson, 2021)	XR	NPO	USA	public-private industries, governments, and academic organisations	Other search
	The IEEE Global Initiative on Ethics of Extended reality (XR) Report: Ethics in Education ( Mangina, 2021)	XR	NPO	USA	public-private industries, governments, and academic organisations	Other search
	The IEEE Global Initiative on Ethics of Extended reality (XR) Report: Extended reality (XR) and the erosion of anonymity and privacy ( McGill, 2021)	XR	NPO	USA	public-private industries, governments, and academic organisations	Other search
	An ethical code for commercial VR/AR applications ( Ramirez *et al.*, 2021)	VR/AR	Academic	USA	Developers and designers	Citation chaining
	Toward a framework for developing virtual reality skills training in human services ( Davis *et al.*, 2021)	VR	Academic	USA	Educators and workforce administrators	
**2022**	The IEEE Global Initiative on Ethics of Extended reality (XR) Report: Metaverse Governance ( Stephens, 2022)	Metaverse	NPO	USA	Public-private industries, governments, and academic organisations	Other search
	The IEEE Global Initiative on Ethics of Extended reality (XR) Report: Diversity, inclusion, accessibility ( Fox & Thornton, 2022)	XR	NPO	USA	Public-private industries, governments, and academic organisations	Other search
	Life, the Metaverse and Everything: An Overview of Privacy, Ethics, and Governance in Metaverse ( Fernandez & Hui, 2022)	Metaverse	Academic	China	Developers and designers	Scopus/IEEE
	Markkula Center: A Code of Ethics for the Metaverse ( Heider, 2022)	Metaverse	Academic	USA	Unspecified	Other search
	Responsible Design and assessment of a SARS-CoV virtual reality rehabilitation programme: guidance ethics in context ( Smits *et al.*, 2022)	VR	Academic	Netherlands	Researchers	Scopus/IEEE
	Ethical Principles for the Metaverse ( Ministry of Science and ICT, 2022)	Metaverse	Government agency	South Korea	Individuals and organisations in the private and public sectors, such as companies, academia, research institutes, civil society, and the government	Other search
	XRSI Recommendations for the Biden- Harris Administration ( Pearlman, 2022)	XR	NPO	International	Public-private industries, governments, and academic organisations	Other search
**2023**	XR and General Purpose AI: from values and principles to norms and standards ( Adomaitis & Grinbaum, 2023)	XR	Consortium	EU consortium	Unspecified	Other search

### Synthesis of results

The analysis of the data, elaborated under Results, was accomplished by SC while the discussion section was written by SC, RdcB, and RB. SC, RdcB and RB reviewed, proofread, and revised the document. Direct quotes are used throughout the results section to support the identified themes thereby enhancing the credibility of the qualitative reporting and validation process. The identified principles were explicit and directly extracted from the documents because of the normative nature of the selected documents. Those that were less explicit were identified and coded based on the coder/reviewers’ interpretation of the authors’ intended meaning. As stated earlier, the organization of principles within categories of values occurred over time during discussions among reviewers (authors) on how to best present the data. The process was iterative.

## Results

### Selection and characteristics of sources of evidence

**Figure 2.**
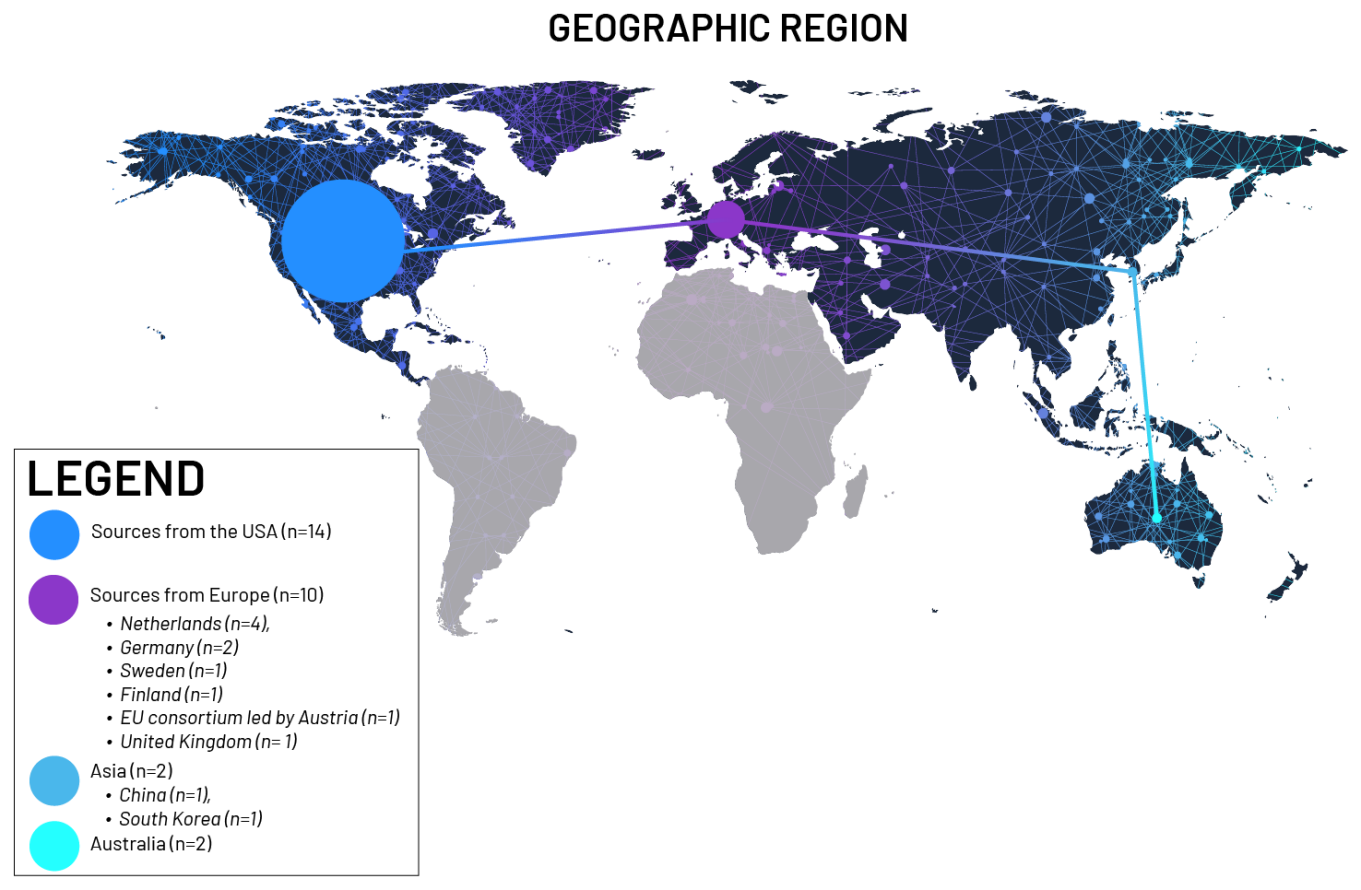
Geographic distribution of publications based on location of authors. (Graphic created by 2 ^nd^ author Alina Kadlubsky)

Twenty-eight (28) documents were identified for coding and content analysis (See Table 3) ( Cox *et al.*, 2024). These documents note ethical issues and suggest frameworks, guidelines, recommendations for codes of conduct and best practices for immersive technologies. Fifty percent (50%) of the papers were written/issued by US-based researchers or organisations. Thirty-six percent (36%) were from Europe, and the remaining from Asia and Australia ( Figure 2).

- Sources from the USA (n = 14; 50.0%),
- Sources from Europe (n = 10; 35.7%):
  - Netherlands (n = 4), Germany (n = 2), Sweden (n = 1), Finland (n = 1), EU consortium led by Austria (n = 1), United Kingdom (n = 1)
- Asia (n = 2; 7.1%): China (n = 1), South Korea (n = 1)
- Australia (n = 2; 7.1%)

**Figure 3.**
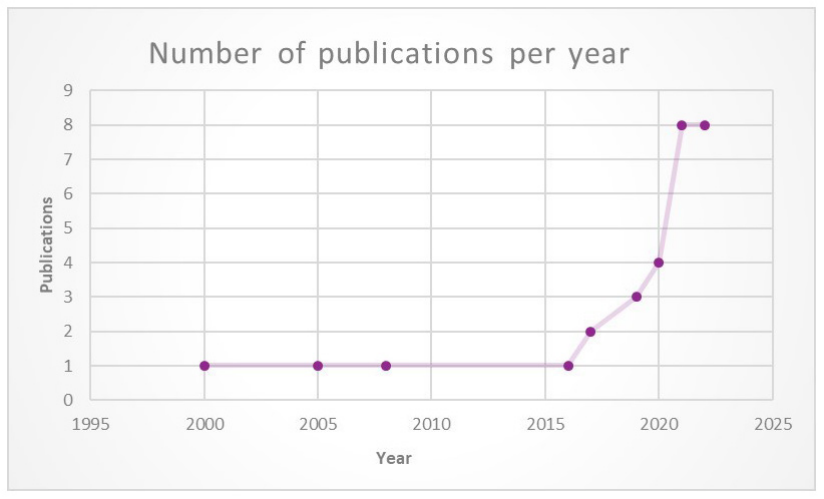
Number of publications regarding ethical frameworks for immersive technologies over 20 years. (Graphic created by 1 ^st^ author Shereen Cox).

Approximately 70% (n = 20) of the papers were issued between 2020 and 2023, of which 17 were in 2021–2022 ( Figure 3). The main authors were academics and non-profit organisations. The target audience was predominantly researchers, industry practitioners and educators. Twelve (12) of the papers were general in addressing issues related to various types of immersive technologies, while five (5) focused only on experiences with VR, four (4) focused exclusively on the Metaverse, two (2) focused on both VR and AR, and one (1) addressed issues related to AR only.

**Figure 4.**
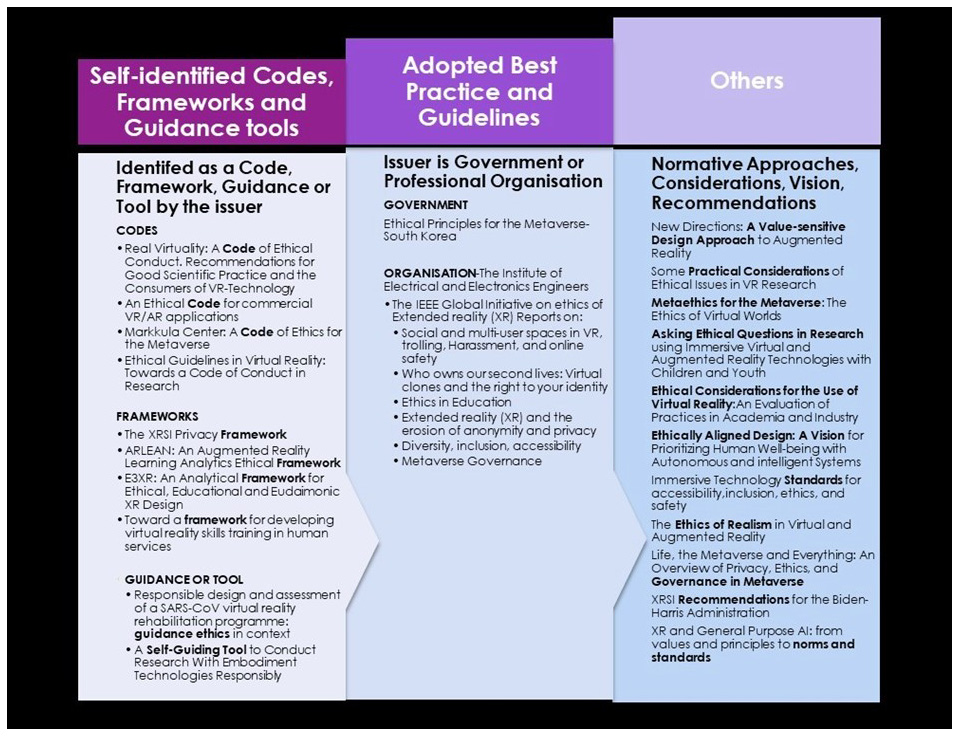
Categorisation of analysed documents.

Ten papers were identified by their authors as either a code (n = 4), a framework (n = 4), or guidance/tool (n = 1 each). Seven papers were identified as best practice standards or guidelines issued by governments and a professional organisation ( Figure 4). Eleven were categorised as others because of the variation in titles, including terms such as approaches, considerations, and vision for the various types of immersive technologies. The main target audience were practitioners in health conducting research on the impact of virtual reality in psychology and children, industry practitioners (such as developers and designers), policymakers, and educators.

### Synthesis of results - Thematic categories

Three (3) thematic categories were noted for ethical issues ( Table 4), and seven (7) thematic categories were coded for foundational values with 22 corresponding principles ( Table 5). Some characteristics classified under principles are virtue-based e.g, honesty, responsibility. However, this classification was made solely for practical reporting purposes and not intended to conflate virtue-based ethics with principle-based ethics from a theoretical perspective. The approaches to the identified ethical issues were organised according to the identified responsible stakeholders. Below we will first elaborate on the ethical issues, followed by an elaboration of the foundational values and the corresponding principles. Lastly, we elaborate on the approaches to the identified ethical issues. 

**Table 4.** Summary of ethical issues.

CATEGORIES FOR ETHICAL ISSUES	CODES
**Issues related to use of Data and ** **Technology**	**Breach of data privacy and confidentiality** **Informational, volumetric, and physical breaches** • Constant data capture without consent • Data can be decoded and identified **Obfuscation of data** • Compromised informed consent **Misuse of data** • Surveillance • Deception • Indoctrination
	**Navigating real and virtual lives** • Embodiment and re-entry problems • Manipulation or interaction with avatars • Individuals' awareness of Metaverse • Avatars with self-awareness • Development of tech without ethical reflection • Dual or unintended use of technology • Attention distraction **Trust** • Controlled social interactions • Nudges
**Issues related to Human Rights -** **Societal & Individual**	**Respect for persons and dignity – Society** **Issues of discrimination, inclusivity and diversity** • Persons with disabilities • Marginalised populations • Elderly **Inequity** • limited or no access to XR technologies **Lack of representativeness in XR** • Sense of belonging **Impact on vulnerable groups** • Mental incapacitated • Disabilities • Children and youth • Elderly
	**Respect for persons and dignity – Individual** **Harm – Physical or psychological, and emotional** • Safety, harassment, exploitation • Addiction, hyper reality-mental health consequences due to AR content, virtual violence **Ownership and Individuation, Identity** • Ownership of second lives • Ownership in the metaverse-avatars • Virtual identity and concept of personal space **Loss of agency** • Deception, therapeutic misconception • Virtual identity • Autonomy and consent
**Issues related to Society, Economy ** **and Environment**	**Negative impact on society** **Issues of social inclusivity and user acceptance** **Sustainability and concern for future generations** **Criminal and anti-social behaviours – Cybercrimes** Fraud Identity theft Sexual harassment Hacking **Impact on economy** • Monetisation **Lack of or insufficient governance** • Jurisdiction • Ownership and property issues

**Table 5.** Summary of foundational values and principles in immersive technologies.

VALUES (7)	PRINCIPLES (22)	
**RESPECT FOR PERSONS**	Freedom Informed consent Inclusion Right or ability to withdraw or stop Privacy Confidentiality Tolerance
**WELL-BEING**	Beneficence Prosperity
**SAFETY**	Non-maleficence Equivalence
**INTEGRITY & TRUST**	Accountability Fidelity Honesty Responsibility Sincere identity Transparency
**JUSTICE**	Accessibility Equity Reciprocity
**RESPONSIVENESS**	Anticipation Adaptivity

### Ethical issues

***Issues related to data and use of technology.*** The main issues related to data are concerns regarding breach of privacy and confidentiality, the volume of data extracted, and lack of clarity in language or jargon when information is shared ( Fernandez & Hui, 2022; Madary & Metzinger, 2016; McGill, 2021; Pearlman, 2020; Ramirez *et al.*, 2021; Slater *et al.*, 2020; Southgate *et al.*, 2017). Data breaches may be informational, volumetric, or physical ( Christopoulos *et al.*, 2021; Mangina, 2021; McGill, 2021; Southgate *et al.*, 2017). Other relevant concerns are constant data capture without consent, the possibility of data being decoded and made identifiable, and data misuse. Data obfuscation makes data incomprehensible based on the technical language used and the volume of data to process ( Fox & Thornton, 2022), and may preclude true informed consent. Data misuse include surveillance, deception, and the possibility of covert indoctrination based on information feeds. Issues related to ensuring data privacy and consent concerning immersive technologies featured significantly across all papers.

One of the main issues regarding the use of XR technology is the difficulty with navigating real and virtual lives/experiences ( Behr *et al.*, 2005; Luro *et al.*, 2017; Madary & Metzinger, 2016; The IEEE Global Initiative on Ethics of Autonomous and Intelligent Systems, 2019). Users of immersive technologies may experience physical world re-entry problems due to issues related to virtual embodiment ( Behr *et al.*, 2005; Lee & Hu-Au, 2021; Luro *et al.*, 2017; Madary & Metzinger, 2016; Slater *et al.*, 2020). Immersive technologies may also incorporate nudges and control of the social interactions of individuals in the virtual space ( Eriksson, 2021; Madary & Metzinger, 2016; Ramirez *et al.*, 2021; Slater *et al.*, 2020; The IEEE Global Initiative on Ethics of Autonomous and Intelligent Systems, 2019). Re-entry problems due to embodiment and controlled interactions may significantly impact users, but most especially vulnerable users such as children and participants in psychological research ( Madary & Metzinger, 2016; Slater *et al.*, 2020; Southgate *et al.*, 2017). The authors note several physical and psychological manifestations of re-entering physical reality from a virtual experience such as motion sickness, postural stability, cognitive disturbances, and information overload ( Madary & Metzinger, 2016; Ramirez *et al.*, 2021). One author noted that re-entry problems and embodiment might manifest both positively and negatively ( Ramirez *et al.*, 2021). However, it is important to emphasise that the main concern with re-entry and embodiment is the possible compromised sense of agency due to the cognitive, emotional, and behavioural disturbances/challenges when returning to reality after immersive (virtual) experiences ( Madary & Metzinger, 2016; Ramirez *et al.*, 2021; Slater *et al.*, 2020; The IEEE Global Initiative on Ethics of Autonomous and Intelligent Systems, 2019).

Certain virtual environments, especially embodiments in VR, can induce change of one's attitudes and behaviour during or after participating in VR experiments ( Madary & Metzinger, 2016). Changes of perception after re-entry into the physical world also affect decision making by the participant due to a false sense of loss of agency ( Madary & Metzinger, 2016), and the researcher must be aware that these effects can infringe the autonomy of the participant ( Bliznyuk, 2019). Cognitive, emotional, and behavioural disturbances after re-entry into the physical world following VR experience were described for VR, though they are valid for AR as well ( Slater *et al.*, 2020).

Dual or unintended use of technologies is another ethically relevant concern.

Dual use is a well-known problem in research ethics and the ethics of technology, especially in the life sciences. Here, we use it to refer to the fact that technology can be used for something other than its intended purpose, to (sic) military applications. In the context of VR technology, one will immediately think not only of drone warfare, teleoperated weapon systems, or "virtual suicide attacks," but also of interrogation procedures and torture. It is not in the power of the scientists and engineers who develop the technology to police its use, but we can raise awareness about potential misuses of the technology as a way of contributing to precautionary steps ( Madary & Metzinger, 2016, p. 10)

While the foreseeability of unintended use of technology may be a challenge, developers ought to consider the possibility and not only focus on the intended use but exercise due diligence in preventing harm.

We must consider beforehand the potential harms of virtual reality to ensure that such technologies are neither intentionally nor accidentally misused. Accounting for these new forms of harm protects both the users and developers of VR/AR, who are both fundamental to the progression of good VR and AR technology ( Ramirez *et al.*, 2021, p. 4).

***Issues related to human rights.*** The ethical issues in relation to human rights in virtual spaces were discussed in the wider societal and individual contexts. At the societal level, authors note concerns regarding discrimination against vulnerable and marginalised populations such as those with mental illness and children. They also note the lack of access and representativeness for persons with varying levels and types and degrees of disabilities. Others note that users of immersive technologies may experience a sense of not belonging. This was especially in relation to users of the Metaverse ( Fernandez & Hui, 2022; Ministry of Science & ICT, 2022). At the individual level, immersive technologies may have negative effects on users. The main ethical challenge is in relation to harm. Harm may be physical, psychological, and/or emotional. Physical manifestations of harm include motion sickness (nausea, vomiting, disorientation, palpitations) ( Behr *et al.*, 2005; Madary & Metzinger, 2016). Psychological or emotional harm manifests as depression, confusion, trauma, and aggressive behaviours such as game rage ( Behr *et al.*, 2005; Madary & Metzinger, 2016). The risk of psychological harm due to long-term effect of immersion is another ethically relevant issue ( Lee & Hu-Au, 2021; Madary & Metzinger, 2016; Southgate *et al.*, 2017).

Mention was also made of identity challenges and ownership of virtual space ( Fernandez & Hui, 2022; Stephens, 2022). Users of immersive technologies may assume a virtual identity that is different from their physical identity, for example, the creation of virtual clones and avatars that may or may not physically or in other ways resemble or reflect the actual user. The risk of identity theft is an essential ethical and legal issue that needs to be addressed. A proposed solution is the right to ownership of identity and, by extension, virtual clones and avatars ( Eriksson, 2021; Stephens, 2022).

Loss or compromised sense of agency was identified as an ethical issue. An example of compromised agency is risk of depersonalization disorder with some VR applications due to the generation of “illusory feelings as if the virtual world is real” ( Madary & Metzinger, 2016). Agency may also be lost when deception tactics are employed in the virtual world or gaming or research.

***Issues related to society, economy, and the environment.*** Concerns regarding the negative impact of increased use of immersive technologies on society were pervasive, especially in the papers on the Metaverse ( Fernandez & Hui, 2022; Stephens, 2022). Social inclusivity and sustainability, especially regarding future generations, are important considerations for XR development. Additionally, concerns were expressed about the possible increase in crime (real or virtual) with the use of XR technologies. Crime is discussed in the context of whether a violation in the real/physical world would be considered a crime in the virtual world. For example, whether sexual or other forms of assault/violence on an avatar would be tantamount to sexual harassment, rape or assault in the real world. However, for the more explicit cybercrime acts such as fraud, identity theft, stalking and hacking, several authors raised concerns about how this may impact on society as noted in the text excerpts below.

Crimes associated with XR are more difficult to penalise. It depends on where it occurred (XR, hybrid, or real world) and where the impact occurred (XR, hybrid, or real world) ( Stephens, 2022, p. 18).
  XR could be used to inflict psychological harm upon the user in the form of harassment, bullying, torture, hate speech or assault. With its strong aspects of graphic and haptic realism, embodiment and presence, it could induce trauma and exacerbate violations of personal space such as simulated touching or grabbing ( Lee & Hu-Au, 2021, p. 4).
  The depiction or embodiment of many kinds of immoral acts are possible in XR, including graphic acts of violence, torture, rape, robbery, and grand theft. These kinds of realistic depictions can lead to trauma, stress or anxiety ( Lee & Hu-Au, 2021, p. 9).

Monetisation and lack of or insufficient governance were also concerns as they are considered incentives to capture a wider audience ( Lee & Hu-Au, 2021; Stephens, 2022). The monetisation of the Metaverse may inadvertently impact society's wider economy as it may “fuel global competitiveness and increase regulatory pressures as countries try to safeguard and extend their domain to XR spaces, leading to unanticipated public-private partnerships that will create new country alliances or lead to the breakdown of others” ( Stephens, 2022, p. 9). Attention monetisation, i.e., models that foster dependency and aim to maximize user attention for profit” is addiction dependent and may lead to wasted time, money, and loss of agency ( Lee & Hu-Au, 2021).

### Foundational values and corresponding principles

Thematic analysis yielded seven distinct theme categories for values and 22 principles. The process of connecting principles to relevant values was iterative. Eventually, there was consensus on the main values and related principles as represented in Table 5. The values are: 1) Respect for persons, 2) Well-being, 3) Safety, 4) Integrity, 5) Trust, 6) Justice, and 7) Responsiveness.

**Figure 5.**
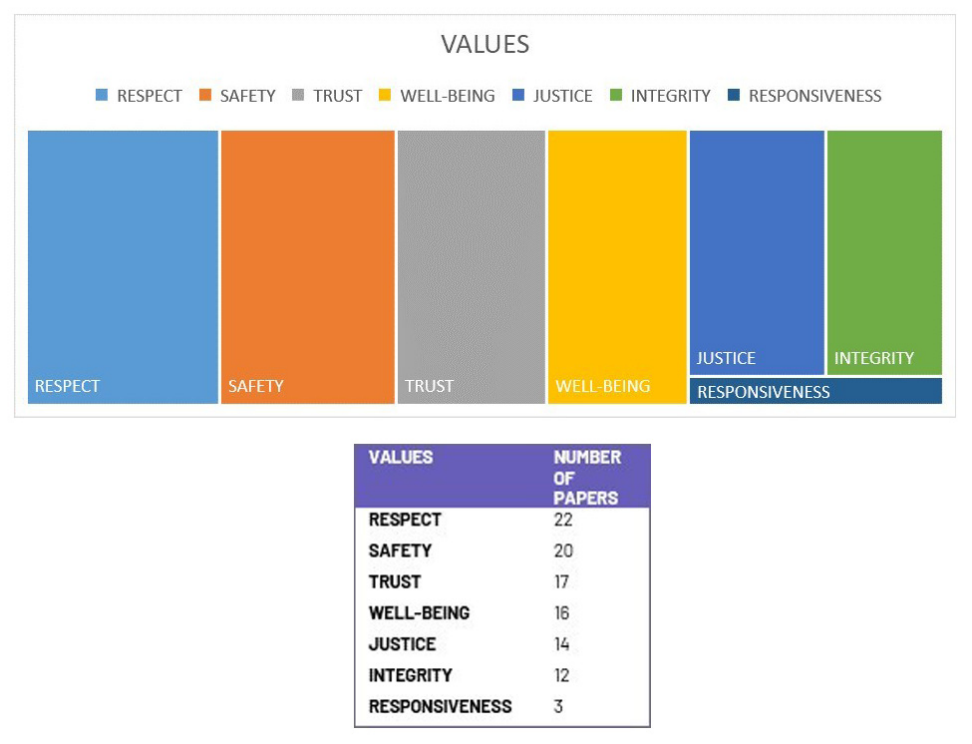
Distribution of values. (Graphic created by 1 ^st^ author Shereen Cox)

The most prominent values featured in more than 70% of the reviewed documents are respect, safety, and trust. Well-being and integrity featured in more than 50%. Responsiveness was the least featured value ( Figure 5).

### Foundational values

***Respect for persons, well-being, and safety.*** Respect for persons is considered a fundamental human right, while human well-being should be the centre of the development and use of immersive technologies ( Aymerich-Franch & Fosch-Villaronga, 2020; Behr *et al.*, 2005; Luro *et al.*, 2017; Madary & Metzinger, 2016; Mangina, 2021; Southgate *et al.*, 2017).

Safety was discussed in the context of avoiding or minimising harm and risks in developing and using immersive technologies ( Aymerich-Franch & Fosch-Villaronga, 2020; Behr *et al.*, 2005; Christopoulos *et al.*, 2021; Lee & Hu-Au, 2021; Luro *et al.*, 2017). A safe virtual environment is fundamental to trust and a positive user experience ( Behr *et al.*, 2005; Bliznyuk, 2019; Lee & Hu-Au, 2021; Ramirez *et al.*, 2021; Smits *et al.*, 2022). The principles connected to safety include non-maleficence and equivalence ( Bliznyuk, 2019; Ramirez *et al.*, 2021). Safety warnings featured as a recommendation for users of headsets, especially for the more vulnerable users such as children ( Luro *et al.*, 2017; Mangina, 2021; Ramirez *et al.*, 2021).

***Integrity and trust.*** Integrity and trust were noted as important values for consumers/users of technologies. Integrity was interpreted as ensuring that regulatory frameworks and industrial standards were in place to facilitate public trust. One author notes the following:

Trust matters. It allows us to reveal vulnerable parts of ourselves to others, and to allow us to know others intimately in return. Moreover, on the societal level trust enhances our social capital. Thus, because augmented reality often supports interactions among persons –particularly interactions that have the potential to leave some persons vulnerable to the actions of other persons – it becomes crucial to design augmented reality such that trust can thrive ( Friedman & Kahn, 2000).

Integrity was discussed in terms of both scientific processes and persons. Scientific integrity refers to designing systems that do not deceive or manipulate users. In one paper the authors note:

XR designers sometimes incorporate strategies that deceive the user, hide the true intent of an interface element, or ultimately guide a person to do what they do not necessarily wish to do, or actions that may not be in their best interest. For instance, “dark UX patterns”–carefully designed techniques such as bait and switch, hidden cost, or misdirection–have been criticised as unethical to users ( Lee & Hu-Au, 2021).

Individual integrity is important in relation to use of sincere identities ( Eriksson, 2021; Ministry of Science and ICT, 2022).

***Justice and responsiveness.*** Justice was discussed largely within the context of fairness, equality, and freedom from biases ( Behr *et al.*, 2005; Bliznyuk, 2019; Friedman & Kahn, 2000; Lee & Hu-Au, 2021; Luro *et al.*, 2017; Madary & Metzinger, 2016; Slater *et al.*, 2020; Southgate *et al.*, 2017; Stephens, 2022). The development of technologies should be such that it “avoid(s) a situation where only privileged members of society benefit from technological advances” ( Madary & Metzinger, 2016). The principles aligned to the attainment of justice are accessibility, equity, and reciprocity. To this end, researchers, developers, and designers are implored to ensure that their systems should anticipate and mitigate against biases ( Boyd-Noronha, 2020).

### Corresponding principles

**Figure 6.**
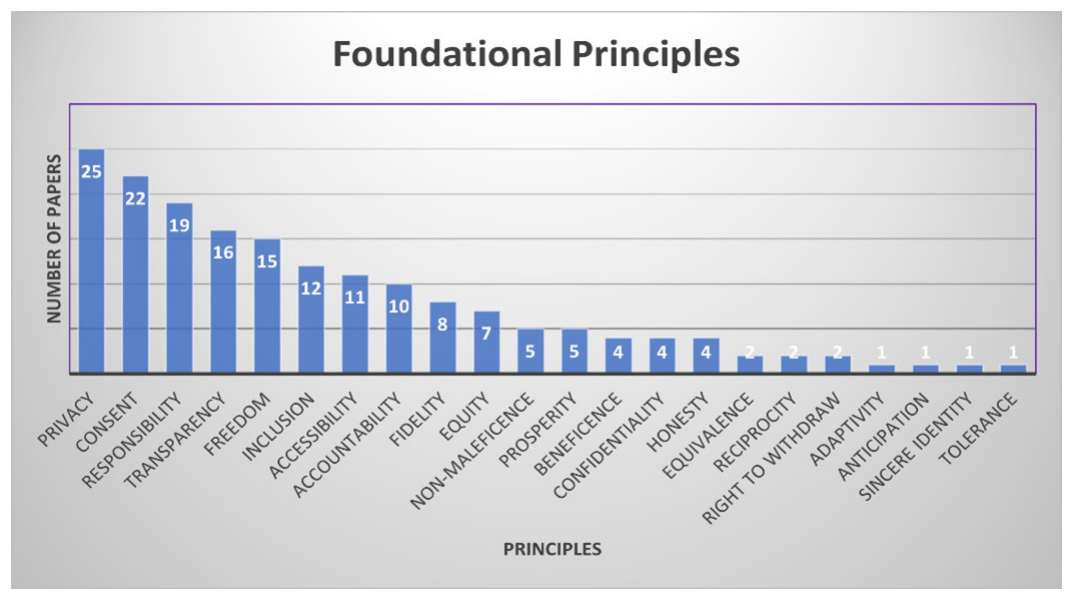
Distribution of principles. (Graphic created by 1 ^st^ author Shereen Cox).

With the values, the documents also identified accompanying principles. The dominant principles are privacy, informed consent, responsibility, transparency, and freedom, which featured in approximately 50% of the reviewed documents ( Figure 6). Privacy, freedom, and informed consent are foundational in respect for persons or users of immersive technologies. Transparency and responsibility refer to the integrity of the technologies and the developers.

***Privacy, confidentiality, consent and freedom.*** Privacy and informed consent to the use of personal data are the most fundament principles in the development of XR technologies ( Fernandez & Hui, 2022; Friedman & Kahn, 2000; Madary & Metzinger, 2016; Pearlman, 2020; Ramirez *et al.*, 2021; Southgate *et al.*, 2017). The right to privacy and by extension non-interference is considered a human right ( Aymerich-Franch & Fosch-Villaronga, 2020). Confidentiality is a term closely aligned to protection of privacy ( Boyd-Noronha, 2020).

The right to privacy is the right to one's identity in any form (including name, image, voice, preferences) remaining private, that is, not becoming publicly disclosed [...] it is crucial to maintain the right to privacy of individuals given that disclosure of private information may be seriously harmful to the psychological well-being and social standing of the affected person ( Slater *et al.*, 2020, p. 6).

Freedom, in the documents, referred to non-manipulation and freedom of choice ( Aymerich-Franch & Fosch-Villaronga, 2020; Fernandez & Hui, 2022; Luro *et al.*, 2017; Madary & Metzinger, 2016; Boyd-Noronha, 2020; Fernandez & Hui, 2022; Ramirez *et al.*, 2021). Freedom was closely associated with references to respect for autonomy and dignity ( Behr *et al.*, 2005; Boyd-Noronha, 2020; Fox & Thornton, 2022; Luro *et al.*, 2017; Madary & Metzinger, 2016; Smits *et al.*, 2022). According to IEEE, "agency is the capacity of individuals to act independently and to exercise free choice, a quality fundamental to democratic ideals" (IEEE: Ethically aligned Design). Loss of a sense of agency through manipulation and use of algorithms that foster dependence is considered an infringement of freedom and autonomy ( Behr *et al.*, 2005; Adomaitis & Grinbaum, 2023; Madary & Metzinger, 2016; Smits *et al.*, 2022).

Business models that foster dependency and aim to maximise user attention for profit are unethical, as numerous studies have shown correlations with decreased quality of life, depression, anxiety, and other impacts on mental, physical, or social health. In contrast, XR designs could reject such algorithms and instead consider how to provide greater freedom and autonomy, self-regulation, and connection with others ( Lee & Hu-Au, 2021, p. 4).

***Fidelity, responsibility and transparency.*** Fidelity, Responsibility and Transparency are noted as integral principles to maintain trust in immersive technologies. Developers and users are expected to be responsible in development and conduct.

Agents who bear responsibility for virtual actions include the developer, the "trainer" (overseeing the selection of training data), the manufacturer, and the user. In each case, the sharing of responsibility should be determined depending on context ( Adomaitis & Grinbaum, 2023, p. 3)

***Non-maleficence (avoiding harm), equivalence and the right to withdraw/stop.*** Although not numerically significant in terms of the use of the explicit term "non-maleficence" in the reviewed documents, indirect reference was made to the synonymous term "avoiding or preventing harm" ( Behr *et al.*, 2005; Boyd-Noronha, 2020; Adomaitis & Grinbaum, 2023; Luro *et al.*, 2017; Madary & Metzinger, 2016; Smits *et al.*, 2022). As discussed earlier, harm may be physical, psychological, or emotional. To this end, all principles are considered of significant relevance despite frequency of occurrence in texts. Two authors addressed non-maleficence using the equivalence principle ( Bliznyuk, 2019; Ramirez *et al.*, 2021). This principle is presented as rule of thumb for comparing harms in the physical and the virtual worlds. The Equivalence Principle states:

If it would be wrong to allow a person to have an experience of something in the real world, then it would be wrong to allow a person to a virtually real analogue of that experience. As a simulation's likelihood of inducing virtually real experiences in its subject increases, so too should the justification for the use of the simulation ( Ramirez *et al.*, 2021, p. 8).

The right to withdraw or stop participation in a VR experience is stressed as particularly important for users. In the context of research using VR, one author notes that:

Participants should be instructed how to quit the VR experience and to deposit the equipment in case of too intense emotions or the feeling of being stressfully overwhelmed by the virtual environment. During the procedure, an experimenter should be available in the laboratory who can assist the participants to terminate exposure and re-enter reality if severe discomfort, information overload, or anxiety arise ( Behr *et al.*, 2005, p. 672).

***Inclusion, accessibility, tolerance and equity.*** Inclusion and accessibility are principles aligned with the right to non-discrimination. These principles are noted as relevant to protecting fundamental human rights of respect for persons and dignity. The IEEE and the Cyber XR coalition emphasise the importance of "leaving no one behind" in the development of immersive technologies. Emphasis is placed on inclusion and access for persons with disabilities and tolerance for persons with varying representations (physical, cultural, and otherwise) in the virtual space. One author highlights an already existing digital divide in society.

Certain groups of people already face the digital divide: women, the poor, the elderly, and the disabled. Not only does this gap need to be closed, but continuous Education will also be needed as new technologies are deployed and new economies developed ( Stephens, 2022, p. 19).

### Approaches to ethical issues

To approach the ethical issues earlier identified, the reviewed documents provided recommendations to society/governance structures, industry, research performing organisations, and individuals. The ethical principles above were frequently cited as reasons for the recommendations.

***Society/governance.*** Within the societal domain, the ethical issues that would be addressed are related to data, technology, and human rights. Many authors referenced data protection and civil/human rights laws and conventions. Recommendations are made for applying existing laws to immersive technologies and the subsequent enforcement of these laws. These recommendations note the need to protect privacy, prevent data misuse, ensure consumer rights, protect children, and prevent discrimination ( Luro *et al.*, 2017; Madary & Metzinger, 2016; Ramirez *et al.*, 2021; Southgate *et al.*, 2017). Another important step would be the official recognition of virtual identity. Recommendations were made in the context of applying copyright laws to virtual identities, particularly virtual clones ( Eriksson, 2021; Stephens, 2022). Other areas to be addressed would be ownership of virtual space concerning property and avatars ( Eriksson, 2021; Fernandez & Hui, 2022; Stephens, 2022). Guidelines are recommended for addressing economic disparities and the possibility of exploitation. Concerns related to virtual crimes could be addressed by examining what should be considered a crime in the virtual space and applying relevant cybercrime laws. Jurisdiction would need to be clarified ( Slater *et al.*, 2020; Stephens, 2022).

***Industry.*** In addressing the ethical issues related to the use of technology, the authors stressed the importance of an inclusive design, development, and implementation approach ( Boyd-Noronha, 2020; Davis *et al.*, 2021; Fox & Thornton, 2022; Friedman & Kahn, 2000; Southgate *et al.*, 2017). Although there are variations in the approaches presented, the most common theme was that technology developments should be "human-centred, safe, and value-sensitive" ( Boyd-Noronha, 2020; Davis *et al.*, 2021; Fox & Thornton, 2022; Friedman & Kahn, 2000). Suggestions were made for adopting existing codes of ethics or standards and tailoring these to the technologies in use. To this end, several authors presented their recommendations in the forms of self-identified codes of conduct, guidelines, tools, and considerations to developers, researchers, educators, and industry practitioners ( Aymerich-Franch & Fosch-Villaronga, 2020; Bliznyuk, 2019; Lee & Hu-Au, 2021; Madary & Metzinger, 2016; Ministry of Science and ICT, 2022; Smits *et al.*, 2022). The need for a standardised methodology across the industry was noted ( Mangina, 2021). Another relevant recommendation was an interdisciplinary collaboration ( Davis *et al.*, 2021; Eriksson, 2021). Several examples of these laws and codes are listed in Table 6.

**Table 6.** Normative approaches in four domains.

DOMAINS	ETHICAL ISSUES	RECOMMENDED EXAMPLES GIVEN APPROACHES		EXCERPTS FROM DOCUMENTS
**SOCIETY** **- GOVERNANCE**	*Ethical issues* * related to use* * of data and* * technology, and* * human rights*	**Application and** ** enforcement of** ** existing laws to XR** ** technologies** *Data protection and * *privacy laws* *Constitutional* *Consumer rights* *Protection of children* *Anti-discrimination* *Accessibility laws*	General Data Protection Regulations (GDPR), Health Insurance Portability and Accountability Act (HIPAA), California Consumer Privacy Act (CCPA) Children Online Privacy Protection Act (COPPA), Family Education rights and Privacy Act (FERPA), Title VI of the Civil Rights Act of Higher Education, European Accessibility Act, The US Rehabilitation Act, The American Disabilities Act, Convention on the Rights of the Child	AR applications raise concerns about reasonable expectations of privacy in public space, as they cannot only record audio-visual information, but also process and aggregate data about a user's surroundings in real time. This information gathering may present special considerations for bystander privacy, especially when government and law enforcement use the technology. To address potential concerns, the Department of Justice should issue **guidelines for law enforcement development and** ** implementation of AR/VR solutions to ensure they maintain First** ** and Fourth Amendment rights protections for the communities in ** **which they deploy this technology** ( Fox & Thornton, 2022).
		**Governance** ** guidelines**	South Korean Government ethics guidelines for the Metaverse	Developers or operators should be aware that the development and operation methods established today may affect the future Metaverse and thus not misuse the Metaverse. They should try to ensure that the Metaverse becomes sustainable ( Ministry of Science and ICT, 2022).
		**Official recognition of** ** virtual identity**	Copyright, identity card and body right	The capability for (original) individuals to **claim the right to their identity ** **and achieve control over the use of their virtual clones** needs to be strengthened. It is beneficial to formulate this right in a familiar way. This will make it easier to explain and discuss. Therefore, it is suggested that a person right law is formulated based on copyright. Such a law could mimic the two-part structure of copyright, so that there is an “economic right” controlling who can use the identity and a "moral right" controlling the original individuals’ right to be connected to the virtual clone. Until such a law is internationally put in place, developers of mixed reality experiences should use the concept as a guiding principle. Note that an alternative term would be body right ( Eriksson, 2021).
		**Ownership of avatars ** **and virtual space**		**Avatar ownership** will be an important issue for regulatory agencies to consider. There are strong reasons to place restrictions on the way in which avatars can be used, such as protecting the interests and privacy of individuals who strongly identify with their own particular avatar on social networks. On the other hand, these restrictions may prove impractical to implement and may unnecessarily limit personal creative freedom. Regulators must strike a rational balance between these concern ( Eriksson, 2021; Stephens, 2022).
		**Measures to address** ** virtual crimes and** ** jurisdiction**	Jurisdiction	Regarding regulatory challenges, jurisdiction becomes more critical. For example, whose national jurisdiction does a virtual-world crime fall under? ( Stephens, 2022).
**INDUSTRY –** **BEST PRACTICE**	*Ethical issues* * related to use of* * technology*	**Inclusive design, ** **development and** ** implementation**	An Ethical Code for Commercial VR/AR applications-Equivalence principle, Safety by Design, Human rights framework, XRSI Privacy framework. Self-assessment using RRI framework, Value sensitive design, Code of Ethics- Association for Computing Machinery, ISO 26000:2010 on Social Responsibility, Software Engineering Code of Ethics, Standardised methodology Inter-disciplinary collaboration Warnings	If it would be wrong to allow a person to have an experience of something in the real world, then it would be wrong to allow a person to a virtually real analogue of that experience. As a simulation's likelihood of inducing virtually real experiences in its subject increases, so too should the justification for the use of the simulation ( Ramirez *et al.*, 2021).
				RRI promotes reﬂection upon the consequences of the outcomes of technology and fosters the incorporation of such reﬂections into the research and design processes. The ﬁve principles of inclusion, anticipation, reﬂection, responsiveness, and transparency that **deﬁne RRI provide** ** a suitable framework for conducting research and innovating** ** responsibly** in any area of R&I, including embodiment technologies ( Aymerich-Franch & Fosch-Villaronga, 2020).
				**Safety by Design** is all about enabling trust in systems, designs, and data so that organisations can lead to transformational change and innovate with confidence. The need for such an approach is becoming more evident every day ( Boyd-Noronha, 2020).
				**Working together to develop** software allows all parties to bring their expertise to the design; and the **collaborative dialogue** often leads to the identification of issues that neither party would independently observe, resulting in an overall improvement of the work's quality ( Davis *et al.*, 2021)
				**Value-Sensitive Design** is primarily concerned with values that center on human well-being, human dignity, Justice, welfare, and human rights ( Friedman & Kahn, 2000).
**RESEARCH & ** **ACADEMIC ** **ORGANISATIONS**	Ethical issues related to individuals in education and research	**Prior ethics review** ** and longitudinal** ** studies to measure ** **effects**	IRB Protocol review and approval	if there is a possibility of physically or psychologically affecting a research participant, an IRB would require the researcher to have a protocol in place if the participant experiences discomfort during or after the study ( Madary & Metzinger, 2016).
				Longitudinal studies and further research into the psychological effects of long-term immersion are needed ( Madary & Metzinger, 2016).
		**Development of new** ** and application of ** **existing codes of ** **ethics for educators ** **and researchers**	**Existing** Belmont report, Nuremburg Code, the World Medical Association's Declaration of Helsinki, the Belmont Report, APA Code of Ethics, **Proposed** ARLEAN, Real Virtuality: A Code of Ethical conduct, E3XR: An Analytical Framework for ethical educational and Eudaimonic Design, Guidance Ethics	The development of contemporary ethical principles in human research can be traced to the Nuremburg Code, the World Medical Association's Declaration of Helsinki, the Belmont Report, and human rights frameworks such as the Convention on the Rights of the Child ( Southgate *et al.*, 2017).
				The approach to **guidance ethics** in the context we proposed here, is even more suitable for merging design with assessment. In healthcare, it is commonly challenging to involve the right users in the design process and embedding technology design within current healthcare services and protocols. A practice-based guidance ethics approach can support designers to optimally embed user needs and values in technology design ( Smits *et al.*, 2022).
		**Clinical oversight**	Real Virtuality: A Code of Ethical conduct	VR researchers aiming at new clinical applications should work in close collaboration with physicians who may be better situated to make informed judgments about the suitability of particular patients for new trials ( Madary & Metzinger, 2016).
**INDIVIDUALS**	Ethical issues related to individuals on a personal level	**User empowerment**	Warnings, informed consent, able to enter and exit VR	Upon entering any virtual realm, individuals should be provided information about the nature of algorithmic tracking and mediation within any environment. This will allow not only for consent regarding the use of their personal data, but for improved trust between individuals and creators of these environments regarding user experience. This could also include a "serendipity on or off" button allowing a user to express their desire for randomness as well ( The IEEE Global Initiative on Ethics of Autonomous and Intelligent Systems, 2019).
		**Identity**	Sincere identity	it is proposed that members of society aim for sincere identity, safe experience, and sustainable prosperity' in the process of participating in the metaverse ecosystem to maximise its potential. In the Metaverse, individuals should be faithful to the values ( Ministry of Science and ICT, 2022).

***Research and academic organisations.*** Researchers featured as one of the main target audiences, especially concerning health and education ( Aymerich-Franch & Fosch-Villaronga, 2020; Bliznyuk, 2019; Lee & Hu-Au, 2021; Madary & Metzinger, 2016; Smits *et al.*, 2022; Southgate *et al.*, 2017). The use of technology in these fields may cause unintended long-term effects. Several authors recommended that researchers should be guided by the existing normative documents for ethics in research, such as the Belmont Report, the Nuremberg Code and the World Medical Association's Declaration of Helsinki. Reference was made to the Convention on the Rights of the Child for the protection of children ( Davis *et al.*, 2021; Madary & Metzinger, 2016; Slater *et al.*, 2020; Southgate *et al.*, 2017). Examples of immersive technologies in health were in relation to VR experiences, especially for the improvement of psychological well-being. Reference was made to the American and British Psychological Associations' Codes of Conduct along with relevant research ethics guidelines ( Behr *et al.*, 2005; Bliznyuk, 2019; Madary & Metzinger, 2016). Several authors presented unique perspectives and self-identified codes with reference to fundamental normative theories such as computer or technology ethics and deontological or teleological approaches to addressing ethical challenges ( Behr *et al.*, 2005; Friedman & Kahn, 2000; Smits *et al.*, 2022). It was observed, however, that variations may need careful examination for comprehension and completeness in addressing all identified ethical issues. The Institutional Review Board (IRB) or Research ethics committee was acknowledged as having a role to play in assessing the possible harms of using VR in research ( Madary & Metzinger, 2016; Southgate *et al.*, 2017). However, it was not noted whether the IRB has the knowledge or capacity to conduct this type of assessment ( Madary & Metzinger, 2016; Southgate *et al.*, 2017). Clinical oversight or clinicians' involvement in research is another recommendation ( Madary & Metzinger, 2016).

***Individuals.*** User involvement and empowerment were noted as integral to the success of ethically aligned designs ( Behr *et al.*, 2005; Fox & Thornton, 2022; The IEEE Global Initiative on Ethics of Autonomous and Intelligent Systems, 2019). Increasing user awareness of the negative effects of immersive experiences and enabling users to exit VR experiences were noted as important to user empowerment ( Cortese & Outlaw, 2021; The IEEE Global Initiative on Ethics of Autonomous and Intelligent Systems, 2019). Avoidance of data obfuscation and information overload to ensure valid informed consent regarding different types of immersive experiences before users enter a virtual space was emphasised ( Behr *et al.*, 2005; Fox & Thornton, 2022; Luro *et al.*, 2017; Madary & Metzinger, 2016). The use of sincere identities and not engaging in anti-social or criminal behaviours was also noted as a personal responsibility of users ( Ministry of Science and ICT, 2022).

## Discussion

### Summary of evidence

The goal of this scoping review is to identify ethical issues in relation to the use of immersive technologies and the related foundational principles and approaches to addressing these issues. We chose the scoping review methodology because it enables knowledge synthesis from a wide cross-section of literature over time. To this end, we identified 28 documents that self-identified as ethical frameworks, codes, or good practices related to XR from grey and academic literature using a pre-determined search and eligibility criteria. The literature search included documents published between 2000 and 2023 written in English. Thematic analysis generated four categories of ethical issues, six values with 22 accompanying principles and four domains addressed in the various approaches.

We note that authors from the USA and Europe wrote most of the included documents. The authors were representative of academia, industry, government, and professional and non-profit organisations. South Korea is the only identified government organisation that issued ethical guidelines for the Metaverse. It may be relevant to note the lack of or under-representation of authors from Asia, Africa, and South and Central America. This could either be a result of limiting the eligibility criteria to the English language only or an indication that ethical guidance on immersive technologies is predominantly from North America and Europe.

Nevertheless, it may be relevant to note that the 2021 scoping review of AI ethics guidelines, which did not have a similar language restriction, also reflected the under-representation of these geographic regions. Unwittingly, geographic under-representation can lead to bias in findings and can limit the generalizability to these regions as local data and context would not be captured. Although the USA and Europe were noted as the leading authors of the reviewed documents, we did not identify a similar ethics guideline as that issued by the South Korean Government for the Metaverse or the use of specific immersive technologies. However, a non-profit organization, Cyber XR Coalition, made recommendations to the Biden administration. The IEEE, a global organization for technology practitioners, issued several recommendations addressing different areas of ethical challenges with immersive technologies to governments, research, and academic and private organizations. Most of these recommendations were published between 2021 and 2022.

***Dominance of principles-based ethics.*** The different guidance documents have varying lists of principles and values/virtues, with some of the principles and values/virtues more common than others, and it remains to be seen if a consensus is to be reached among the various stakeholders from the various global regions. The values and principles that are less emphasised may also be an indication of areas that need more work. We will be able to see which ethical issues emerge in terms of magnitude and frequency, which may confirm or deny the latter claim.

Aside from the above-mentioned observation, it was also noticeable that all the reviewed documents adopt a principles-based approach to ethical justification. By principles-based approach, we refer to a specific way of doing ethics characterized by the identification of guiding principles/values/virtues, none which hold primacy over the other ( Beauchamp & Childress, 2019). Biomedical ethicists, Tom Beauchamp and James Childress, who presented four arguably universal and co-equal principles that are balanced off against each other in the settling of an ethical question or dilemma ( Beauchamp & Childress, 2019), championed this approach. However, the approach has been criticized for arbitrariness of its procedure for moral justification and the legitimacy of the list of principles, and the same criticism may be lodged to the different guidance documents considering that they all have the same ethical foundation ( Duwell, 2014). This finding may be translated into learnings that must be taken into consideration when drafting an XR guidance document, including the following:

a) That principlism is an intuitively acceptable approach utilized in guidance documents, which may be an indication of a convenient approach for the XR4Human’s guidance document.
b) Considering the limitations of the approach as reflected in the literature, guidance document drafters may wish to include or consider other approaches to doing ethics that prove to be useful not only in the elaboration of the ethical issue at hand but also sufficiently guiding for the intended stakeholders. Our succeeding publications will address the latter issue in preparation for an ethics guidance document for XR development.

### Some gaps in the literature

The reviewed papers that discussed ethical issues and normative approaches in relation to the use of immersive technologies in health predominantly focused on ethical dilemmas pertinent to the field of psychology. The welfare of children using immersive technologies was widely discussed, with children identified as the most vulnerable group. Southgate *et al.* (2017) discussed in-depth some of the possible negative consequences of research on children and youth using immersive technology. The authors emphasized an ethics-in-practice approach, i.e., continued vigilance in research with children. Other authors discussed the possibility of various forms of developmental harm as well as infringement on the privacy of children ( Madary & Metzinger, 2016; Pearlman, 2020; Southgate *et al.*, 2017). It was noted that children are particularly vulnerable because their brains are not "fully developed" ( Madary & Metzinger, 2016; Southgate *et al.*, 2017). There appears to be a gap in terms of unknown long-term consequences on the development of children (physical, psychological, and cognitive). For example, there is limited information related to the eye safety of children. However, the technology, through its simulation of 3D, breaks down a neurological link that operates in natural/physical environments that is essential for eyes and visual function to develop normally (normal development is also preventive of eye disease in later life) ( Pladere *et al.*, 2022). Consequently, if eyes and vision do not develop normally, persons could be excluded from using this technology ( Pladere *et al.*, 2022). Some authors discussed harm-mitigating industry best practices, such as warnings against the use of head mount devices by children under 13 years, the need for adult supervision, and limiting virtual time. Others, such as Southgate *et al.* (2017), did not consider these measures as sufficiently protective against future harm. Applying some existing normative frameworks, such as the Convention on the Rights of the Child and the US Children's Online Privacy Protection Rule (COPPA), were considered relevant to guiding developers ( Pearlman, 2020; Southgate *et al.*, 2017).

One document mentions the use of immersive technologies within the workplace in the context of inclusion ( Fox & Thornton, 2022). This is identified as a gap as there has been an increased use of immersive technologies in workspaces, and attention needs to be placed on the impact (positive or negative) on the users. If the technology is not sufficiently inclusive, then this naturally raises ethical concerns. Current commercially available XR head mounted displays may be comfortable to wear for only about 50–60% of the population ( Pladere *et al.*, 2022). If there is a requirement to use this technology to fulfil daily tasks/obligations, but it is a poor fit and uncomfortable to wear or use for any length of time, or do not accommodate for the necessary prescription correction during use – it will put the worker at a disadvantage, which may be noted as a disability.

Although several texts note a role for ethics committees, no information was shared on the competence or capacity of ethics committees to evaluate immersive technologies. Discussions were limited to the need for a review of research for potential harm to participants and follow-up of research involving children/youth ( Madary & Metzinger, 2016; Southgate *et al.*, 2017).

## Limitations and conclusions

### Limitations

Although the librarian's expertise is essential in conducting a comprehensive search, the choice of databases has its limits. The search was limited to the databases Scopus and IEEE based on prior awareness of the expansive representation of number and various types of widely cited publications (Scopus) and the technical focus (IEEE). Guided by the librarian, we considered that other databases predominantly would increase the number of duplicates and mightnot be necessary. However, we note this assumption could be a limitation. Adopting a scoping review methodology reporting descriptive data combined with qualitative analysis also has inherent limitations ( Arksey & O'Malley, 2005). One such limitation is the possible exclusion of relevant texts based on predefined search terms, and there is a risk of not identifying essential policy documents that may not be indexed to facilitate retrieval using our search procedures. Additionally, because this review is part of a larger project with various work packages of interconnected tasks with related objectives, individual tasks have to be very specific to achieve the intended aim. To remain focused to the specific objectives of this review, we restricted the inclusion criteria to publications that prescribe ethics frameworks, guidelines, and codes of conduct, rather than publications that broadly describe or address general ethical risks. Consequently, publications that focused more generally on outlining specific ethical risks of XR technologies were excluded from this review. There is an inherent limitation in this approach because publications that do not explicitly identify in the titles or abstracts as prescribing an ethics framework – but may actually do so in the body of the text – may have been unintentionally excluded.

Descriptive content analysis is recommended for scoping reviews; however, the authors incorporated thematic analysis for some of the findings reported. Thematic analysis is also subject to bias, although we attempted to minimize this by using several coders, an inductive coding strategy, and regular meetings. Additionally, we limited the search to texts written in the English language. Current best practice requires the development and registering of a protocol before embarking on the actual scoping tasks. However, for practical reasons and time constraints due to project deadlines, we adopted Jobin *et al*.’s pre-tested data screening and extraction method for the scoping of guidelines for artificial intelligence. There may be unidentified limitations to this approach.

### Conclusion

Ethical issues were related to misuse of data, breach of privacy, misuse of technologies, human rights concerns, and wider societal challenges such as exploitation, sustainability, and crimes. Several authors presented approaches, which we have coded and organized into four main domains: Society, Industry, Research/Academic Organizations, and Individuals. Although there are variations in approaches across the documents, the central themes to addressing ethical issues in immersive technologies are hinged on the fundamental values of respect for persons, human well-being, safety, trust and integrity, justice and responsiveness and their accompanying principles. We note that proper governance frameworks, inclusive human-centric technology development, and user empowerment are essential to successfully addressing ethical challenges. Several existing normative approaches were presented in the literature, and new suggestions were made to address unique challenges. Nevertheless, we note gaps that may need to be further explored. The South Korean Government appears to have taken the lead in tendering ethics guidelines at a governmental level ( Ministry of Science and ICT, 2022). There is a disparity in the geographic distribution of authors, indicating a possible underrepresentation in the literature. We also note that health was discussed mainly in the context of psychology. The protection of vulnerable groups, especially children, was noted. However, there are concerns that there is limited knowledge regarding the intermediate and long-term effects of immersive technologies. Though XR is already applied within many industries and fields, it's still an emerging technology poised to mark a transition towards mainstream adoption. This transition is driven not only by more accessible and cost-effective hardware such as wearables or head mounted devices (HMDs), but also by its fragmented infrastructure between services and platforms. Like in other emerging technologies, research and policy development in XR lags behind the increasingly rapid pace of development. This pacing problem, aptly described by Thierer (2018), may account for the comparatively few studies identified during this scoping of ethical issues and proposed guidelines. A recommended approach to addressing the challenges of the pacing problem is the development and application of soft laws such as guidelines, policies, and codes of conducts ( Hagemann *et al.*, 2018).

### Recommendations for moving forward

While conducting this scoping review, several gaps were identified within the proposed ethical frameworks and codes of conduct for immersive technologies. These gaps present avenues for future work. Accordingly, we outline five key recommendations aimed at addressing these to strengthen the ethical foundations of immersive technologies:

1. Development and Application of Comprehensive Frameworks: Lawmakers, regulatory bodies, and industry leaders should collaborate to devise and enforce dynamic laws and guidelines that address the evolving landscape of immersive technologies. These frameworks must prioritize ethical considerations and individual rights, ensuring a balanced progression of innovation and societal values.
2. Adoption of Human-Centred Design Principles: Industry practitioners should embed human-centred design principles, emphasizing inclusivity and accessibility, into their development processes. This commitment will not only enhance user engagement but also ensure broader access and relevance.
3. Inclusion and Protection of Participants: Researchers and developers should adhere to stringent ethical standards, as much as possible emphasizing participants’ inclusion in the entire development process, all the time incorporating and acknowledging established and emerging technology ethics and research ethics principles such as informed consent and protection of participant well-being. This entails clear communication of potential risks and dedicated support and inclusion structures, thereby minimizing adverse impacts and promoting ethical research and development practices.
4. Empowerment of Users: Educating users on digital literacy, privacy controls, and ethical conduct in virtual spaces is imperative. By equipping users with the necessary tools and knowledge, we provide users with the necessary tools for the promotion of responsible virtual conduct.
5. Promotion of Responsible Virtual Conduct: Regulators, industry practitioners, and users are responsible in fostering a responsible and respectful online community, one that enhances the overall quality of virtual interactions. Addressing the challenges of identity and conduct in virtual spaces requires a concerted effort to cultivate a culture of sincerity and accountability. Clear codes of conduct, alongside effective enforcement mechanisms, will safeguard the integrity of virtual communities and encourage constructive engagement.

We advocate for the integration of these recommendations in immersive technology development, deployment and usage. This is to ensure that the advancement of immersive technologies is not only marked by their technical innovation but also by their commitment to respect, inclusiveness, and the welfare of humans and a human-centered design of immersive technologies. It is our intent that these findings will contribute significantly to achieving one of the key goals of the XR4Human project, i.e., establishing a robust code of conduct for developers, organizations, and stakeholders involved in the lifecycle of immersive technologies.

## Ethics and consent

Ethical approval and consent were not required.

## Data Availability

The data for this article consists of bibliographic references, which are included in the References section. Zenodo: A scoping review of the ethics frameworks describing issues related to the use of extended reality. https://zenodo.org/doi/10.5281/zenodo.10640479 ( Cox *et al.*, 2024). Dataset scoping review of ethics guidelines.xlsx Scoping of ethics guidelines PRISMA-ScR-Fillable-Checklist.docx Search Documentation ethical framework XR technology (1).docx XR technology ethical framework.txt Data are available under the terms of the Creative Commons Attribution 4.0 International license (CC-BY 4.0).

## Authors and roles

**1 ^st^ author – Shereen Cox (SC), University of Oslo**

Screening, methodology, full text reading, thematic analysis, writing original draft preparation, visualization, internal review editing, revising of final draft, and manuscript submission.

**2 ^nd^ author – Alina Kadlubsky (AK), Open AR Communications**

Screening, full text reading, thematic analysis, visualization, revising of final draft

**3 ^rd^ author – Jonathan Adams (JA), University of Oslo**

Screening, full text reading, thematic analysis, and revising of final draft

**4 ^th^ author – Ellen Svarverud (ES), University of South-Eastern Norway**

Screening, full text reading, thematic analysis, and revising of final draft

**5 ^th^ author – Rigmor C. Baraas (RB) – University of South-Eastern Norway**

Funding acquisition, project administration, conceptualization, writing and revising of final draft.

**Last author – Rosemarie DLC Bernabe (RdcB), University of Oslo, University of South-Eastern Norway**

Funding acquisition, project administration, work package supervision, conceptualization, screening, thematic analysis, writing - original draft preparation, writing and revising of final draft.
