# Supplementary material for: A scoping review of the ethics frameworks describing issues related to the use of extended reality
Source: Open Res Eur. 2025 Feb 10;4:74. Originally published 2024 Apr 18. [Version 2] doi: 10.12688/openreseurope.17283.2 (PMC11862359; doi:10.12688/openreseurope.17283.2)
Supplement: Peer review history

# Reviewer report: Reviewer response for version 2

**DOI:** 10.21956/openreseurope.21253.r51102

**Published:** 2025-03-07

**Authors:** Abraham Mhaidli

**Affiliations:** 1 The University of Michigan, Ann Arbor, USA

This paper conducts a scoping review of ethics frameworks relating to extended reality (XR). Specifically, they discover 28 papers that propose ethical frameworks, guidelines, or tools and review them to understand what are the ethical issues they raise, what principles guide them, and what recommendations they make.

 The paper finds various common ethical issues they raise (including data privacy, etc.), as well as how many of these papers use principle based ethics. They also found that most of these papers originate from the US and Europe.

 Overall, I found this paper to be a good read and necessary work. I think the methodology done is sound, the conclusions drawn from the data valid, and so I recommend acceptance for the paper.

 There are two small areas of improvement I would liked to see the authors expand upon: however, these are my own preferences, and would not prevent me from accepting the paper.

 The first area of improvement has to do with the inclusion criteria of papers. Specifically, a little more detail on how the search terms chosen and/or vetted to ensure that they provided a good overview of results? There is at least one paper that seems to be missing [ref 1] Ethics emerging: the story of privacy and security perceptions in virtual reality. In Fourteenth symposium on usable privacy and security (SOUPS 2018) (pp. 427-442).), where the abstract specifically states " We [...] contribute a concrete VR developer "code of ethics'', created by developers, for developers." This is me nitpicking -- it is difficult for any scoping review to pick out every single paper that has ever been written. But a bit more information on how the search criteria were established and/or validated (if at all) would have been appreciated. I do appreciate the authors raise this in the limitations section.

 The second area of improvement has to do with the recommendations. They feel a little bit generic (education, companies should act responsibly, etc.) and seemingly disconnected from the findings. Something more interesting would have been to see how to create better ethical frameworks. E.g., after studying 28 ethical frameworks, what is missing? What perspectives or ethical approaches need to be incorporated into existing frameworks and tools? There is a little bit of this already in the 'Some gaps in the literature' section, but more would have been appreciated.

 Having said that, these are minor nitpicky points, and should not stand in the way of indexing. Thus, I recommend accept.

Is the study design appropriate and does the work have academic merit?

Yes

Is the work clearly and accurately presented and does it cite the current literature?

Partly

If applicable, is the statistical analysis and its interpretation appropriate?

I cannot comment. A qualified statistician is required.

Are all the source data underlying the results available to ensure full reproducibility?

Yes

Are the conclusions drawn adequately supported by the results?

Yes

Are sufficient details of methods and analysis provided to allow replication by others?

Yes

Reviewer Expertise:

XR harms

I confirm that I have read this submission and believe that I have an appropriate level of expertise to confirm that it is of an acceptable scientific standard.

## References

[1] : Ethics Emerging: the Story of Privacy and Security Perceptions in Virtual Reality. *Semantic scholar* . 10.13016/M2B853K5P 10.13016/M2B853K5P

# Reviewer report: Reviewer response for version 2

**DOI:** 10.21956/openreseurope.21253.r51034

**Published:** 2025-02-25

**Authors:** Joshua Fisher

**Affiliations:** 1 Ball State University, Muncie, Indiana, USA

I am happy with the clarifications and revisions made by the authors.

Is the study design appropriate and does the work have academic merit?

Yes

Is the work clearly and accurately presented and does it cite the current literature?

Partly

If applicable, is the statistical analysis and its interpretation appropriate?

Not applicable

Are all the source data underlying the results available to ensure full reproducibility?

Yes

Are the conclusions drawn adequately supported by the results?

Yes

Are sufficient details of methods and analysis provided to allow replication by others?

No

Reviewer Expertise:

Augmented reality, virtual reality, and XR community practices

I confirm that I have read this submission and believe that I have an appropriate level of expertise to confirm that it is of an acceptable scientific standard.

# Reviewer report: Reviewer response for version 1

**DOI:** 10.21956/openreseurope.18681.r40512

**Published:** 2024-08-14

**Authors:** Philip Nickel

**Affiliations:** 1 Eindhoven university of technology, Eindhoven, The Netherlands

This article presents the results of a structured literature review regarding extended reality technologies, and uses the results to observe gaps in the literature and make recommendations. The topic and approach are timely and important, as this is a good moment to take stock of the ethical response to these increasingly influential technologies and how they link to future research and policy recommendations.

 The article is highly informative and well structured, and the overview of results will be especially useful to readers who want to locate particular ethical issues in this broad literature and find helpful references for further investigation.

 Despite the strengths of the paper, I have two main critical observations:

 First, although the topic is very timely and important, structured literature reviews tend to be more interesting if there is a specific problem related to the literature or to the development of a field of science, technology, or medicine that the literature review can help to address. In this literature review, I did not discern a problem of this kind. Examples would be a struggle in the literature to reconcile ethical issues at the level of the real world with those present at the level of the virtual or augmented world; or the potential for blurring factual and fictional elements in the virtual or augmented layer present in XR technologies; or the lack of a unified approach to a set of converging technologies. (This is not to say that any one of these reasons really obtains in the literature, they are provided only to indicate the category of motivation that might help provide additional interest to readers.)

 Second, there is some circularity in the reasoning for one of the observations about the literature, given the set-up of the review. The authors conclude on page 21 that principle-based approaches dominate the literature. However, given the search terms used by the authors, with *framework*, *guideline*, *regulation*, *law*, *legal*, *norm*, *code*, *good practice* as required terms, it is not surprising that the results revealed principle-based approaches. I suspect that there are many philosophical and social scientific discussions of extended reality that identify ethical issues outside of a principle-based approach, but would not include those search terms. My guess is that some non-principle-based approaches would be hostile to conceptualizing ethics in terms of these search terms; hence the review never had a chance of encountering them.

 Another related point is that the authors include virtue-based approaches within the category of principle-based ethics. However, that is not a standard classification within ethics; more often, virtue approaches are contrasted with principle-based approaches.

 There were also a few places in the manuscript where I felt I needed more information to understand the structure and reasoning:

- On p. 4 in the paragraph starting “Following the methodology…” it was not clear how the Google search fit in to the overall review methodology. A bit more background would have helped me.
- On p. 5 just after the section heading “Data Charting process” I felt I did not understand what was meant by “self-identified in the title”. To me it seemed like many of the included papers from the subsequent list did not satisfy that description.
- On p. 10, the graphic “Geographic Region” contains dots that do not correspond in size to the regional origin of results. I felt I might have misunderstood the intent behind the graphic.
- On p. 13, I was not sure whether the discrimination against vulnerable and marginalized populations was occurring in the physical world or in the virtual world, i.e., whether it was a barrier to access and inclusion in the technology because of usability, or whether it was a matter of oppressive elements within the virtual world that led to “a sense of not belonging”. Although these are both important, they seem potentially very different in terms of what group is marginalized and what mitigation would look like.
- On that same page, I was unsure why identity challenges and ownership of virtual space were being grouped together.
- On p. 14, it seemed strange to me that respect for persons was grouped together with well-being and safety. These seem like very distinct values, in the sense that one is grounded in autonomy and freedom, and the others are grounded in beneficence.
- On that same page under the subheading “Issues related to society, economy, and the environment” the authors mention “the more explicit cybercrime” acts, apparently contrasting them with the previously mentioned acts of “sexual harassment, rape or assault”. I did not understand the contrast.
- On p. 15, I did not understand the meaning and purpose of the graphical element of Figure 5.
- On p. 17, the authors write that “the use of technology in [the] fields [of health and education] may cause unintended long-term effects.” Is this a point about XR technology, or about technology generally?
- On p. 21, the authors write that reviewed papers on “the use of immersive technologies in health were mainly centred on ethical issues concerning the field of psychology.” I found this formulation confusing, because psychology is about more than just health. Also, the statement is ambiguous in the sense that it could be referring to psychological effects of XR technologies in health applications generally (including those for surgery after-care, physical therapy, or whatever), or it could be referring to XR technologies used for specifically psychological ends.

As a final note, I would have appreciated learning more how the authors transitioned from the overview of results, to the recommendations for moving forward. To me, the list of recommendations seemed somewhat unrelated to what came before. A few words of justification and linkage would be welcome.

Is the study design appropriate and does the work have academic merit?

Yes

Is the work clearly and accurately presented and does it cite the current literature?

Yes

If applicable, is the statistical analysis and its interpretation appropriate?

Not applicable

Are all the source data underlying the results available to ensure full reproducibility?

Partly

Are the conclusions drawn adequately supported by the results?

Yes

Are sufficient details of methods and analysis provided to allow replication by others?

Yes

Reviewer Expertise:

ethics of technology, applied ethics

I confirm that I have read this submission and believe that I have an appropriate level of expertise to confirm that it is of an acceptable scientific standard, however I have significant reservations, as outlined above.

# Response

**Published:** 2025-02-07

**Authors:** Shereen Cox

**Affiliations:** Universitetet i Oslo, Oslo, Oslo, Norway

**Thanks for highlighting this observation regarding the classification of virtue-based approaches within the principle-based categories.**

**We concede to the fact that this is not a standard classification within ethics and could create confusion. The reason for organizing the themes in this manner is strictly for practical reporting purposes.**

**We also acknowledge that the search terms would naturally yield results that would be dominant in principle-based approaches. However, this does not mean that there could not have been discussions within the search terms that might have gone beyond a principle-based approach. Therefore, it is by no means inevitable and therefore still relevant finding, that the ethics frameworks for XR that we found are limited to listing principles**.

**In addition to the Scoping review method which allows for inclusion of grey literature, we adapted Jobin et al approach to identifying ethics framework, guidelines for AI. This method used a google search to identify recent or missing frameworks for AI due to the growing literature on the subject.** **While some papers self-identified in the title, others were prescriptive in the abstract. This section has been edited. **  

**The discrimination described was in virtual spaces - the section has been edited to reflect this change. Similarly, barrier to access and inclusion were connected to usability and avatar representation in virtual spaces specifically the Metaverse.** **The grouping of identity challenges and ownership of virtual space was not intended to convey a particular message. They were only reported together.**

**To the query re explicit crimes and the contrast with other acts such as harassment: some occurrences in XR are still being debated as it is not explicitly identified as a criminal acts eg rape of a virtual person (avatar) as some XR content especially in social  VR and gaming scenarios while some are already well established as cybercrimes such as those financial crimes eg fraud.**

**Regarding the query on unintended consequences of technology -** **This is in relation to XR technology due to its unique immersive nature.**

**Regarding the query on psychological effect -The papers reviewed focused on the topic of psychological impact/effects of using XR technologies and how it may affect overall mental/cognitive well-being. These were not in relation to use cases and applications of the technologies.**

**Finally, the section on how we transitioned from the results to the recommendations have been revised to provide more clarity in version 2**

# Reviewer report: Reviewer response for version 1

**DOI:** 10.21956/openreseurope.18681.r40520

**Published:** 2024-06-27

**Authors:** Joshua Fisher

**Affiliations:** 1 Ball State University, Muncie, Indiana, USA

This scoping review analyzes ethical frameworks and guidelines for XR across various domains, covering literature from 2000 to 2023. Employing a qualitative scoping review approach, it examines existing and proposed ethical frameworks, codes of conduct, and best practices. From 28 papers, the analysis identified seven core values and twenty-two principles, categorized into four normative domains. The researchers conclude by recommending the development of legal guidelines, adopting inclusive design practices, minimizing research risks, empowering users, and promoting responsible and sincere virtual interactions.

 While the paper is informative and valuable for advancing the field, it would benefit from a more detailed explanation of the methodology and results development. Elaborating on how the values, principles, and guidelines were derived would enhance their perceived validity.

 The exclusive use of IEEE and Scopus as sources raises concerns, given that other repositories, particularly in the humanities and social sciences, could provide nuanced discussions on ethics and XR. An explanation for selecting only these sources would clarify this choice.

 Regarding the methodology, the implementation of screening processes remains unclear. Were the eligibility criteria listed in Table 2 utilized during the initial screening on the Rayyan platform or at a different stage? The selection of only three articles from the comprehensive South Korean Metaverse ethical guidelines report also requires justification. Additionally, details on how the PRISMA-ScR was applied to highlight nuances in ethical frameworks, the assessment of study quality, and measures taken to mitigate selector biases would be informative.

 Regarding the Data-charting section, addressing the bias introduced by restricting the review to English-language articles would add depth. The synthesis of results section needs a more expansive introduction. It would be beneficial to discuss whether the researchers sought intercoder reliability. The thematic analysis procedures, including how the researchers developed their codebook and organized principles under various values, need clarification.

 The identification of regional underrepresentation in the Discussion—Summary of Evidence section is commendable. Discussing the impact of this underrepresentation on the ethics presented would strengthen the section. While the AI scoping review faces similar issues, relying on this comparison does not adequately address the implications.

 The recommendations are well-founded and constructive. Nevertheless, expanding the gaps in literature or limitations sections to address overlooked ethical issues, such as representation in XR and community practice ethics, would be advantageous. Although this is my area of research, the absence of discussion on these topics was unexpected.

Is the study design appropriate and does the work have academic merit?

Yes

Is the work clearly and accurately presented and does it cite the current literature?

Partly

If applicable, is the statistical analysis and its interpretation appropriate?

Not applicable

Are all the source data underlying the results available to ensure full reproducibility?

Yes

Are the conclusions drawn adequately supported by the results?

Yes

Are sufficient details of methods and analysis provided to allow replication by others?

No

Reviewer Expertise:

Augmented reality, virtual reality, and XR community practices

I confirm that I have read this submission and believe that I have an appropriate level of expertise to confirm that it is of an acceptable scientific standard, however I have significant reservations, as outlined above.

# Response

**Published:** 2025-02-07

**Authors:** Shereen Cox

**Affiliations:** Universitetet i Oslo, Oslo, Oslo, Norway

Thank you for this comment. We have now expanded the section on how values and principles were derived on page 14. It is unclear what the reviewer means by “and guidelines were derived” since we did not propose any guidelines but examined publications we identified as guidelines, codes, etc. We acknowledge that other repositories could have been considered. We chose Scopus as it is one of the most comprehensive and leading databases for widely cited literature. The librarian also recommended and supported this. IEEE is the industry standards agency for technology literature. However, we have now included this limitation.  The eligibility criteria was utilized both during the abstract screening and the full text reading. This is outlined on under Data charting process - synthesis and coding strategy. All sections from the document, Ethical Principles for the Metaverse, published by the South Korean government were coded. This included the text from all three values (sincere identity, safe experience, and sustainable prosperity) as well as all eight practical principles ( authenticity, autonomy, reciprocity, respect for privacy, fairness, personal information protection, inclusiveness, and responsibility for the future).  Admittedly, we did not adhere strictly to the PRISM-Scr guidelines as we noted in the methods, we applied Jobin et al’s method for identifying ethics guidelines for AI. However, based on best practice, we did our best to minimize bias by having several reviewers (3) for the initial screening – when there was disagreement, we had an independent reviewer to help with final decision. We also met weekly to discuss codes and differences in our interpretation of identified principles. If the reviewer is asking whether we assessed the quality of the studies included, we did not assess quality of the papers as this was not one of our core objectives and is not usually a task in scoping reviews. A scoping review does not involve a formal assessment of the quality of included studies. The primary goal of a scoping review is to provide a broad overview of the existing literature on a particular topic, regardless of the quality or strength of the research. However, we also acknowledge the inherent weakness of not doing so. If the reviewer is asking about the quality of our scoping, we sought to follow the step wise process outlined by Arksey and O Malley combined with the already The  validated method of Jobin et al. We also had two independent internal reviewers for the XR4Human project examine and give comments on  the paper before we submitted. We acknowledged the language in the Limitations section. This inadvertently would impact on the comprehensiveness of the study in that there may be other relevant papers in languages that were not identified nor reported See revised Limitation.  We sought to achieve intercoder reliability during our weekly meetings to discuss the identified codes. The thematic procedure evolved because of the a priori knowledge of coders(reviewers) in ethics and technology. Some of the identified principles were explicit and directly extracted from the documents as these were mainly normative documents. Those which were more implicit emerged and were coded based on the coder/reviewers’ interpretation of what the authors meant. The organization of principles under values was derived during discussions among reviewers(authors) on how to best to present the data. The process was iterative. This is somewhat subjective. We have now expanded this section to note that some implications of geographic underrepresentation would lead to bias in findings and limits the generalizability to these regions as local data and context would not be captured. Whilst addressing perceived overlooked ethical issues could be considered, there will still remain some bias in any attempt to identify overlooked ethical issues as this would assume that we as reviewers had comprehensive a priori knowledge of these. We noted some overlooked issues in the gaps in the literature section, however we used the word some to highlight that we are aware that we do not know all the possible gaps.

# Reviewer report: Reviewer response for version 1

**DOI:** 10.21956/openreseurope.18681.r40516

**Published:** 2024-06-08

**Authors:** Mark McGill

**Affiliations:** 1 University of Glasgow, Glasgow, Scotland, UK

The article examines existing ethical frameworks and guidelines targeting extended reality technology, and through qualitative analysis distils them into the common fundamental values and principles being discussed. Such work is necessary, and provides insight not just into said values/principles, but also crucially in the gaps here - that there is a patchwork of literature that tries to tackle the ethics of XR, but ultimately lags behind research into specific emerging and anticipated risks/harms, meaning as-yet there is no one framework (or even collection of frameworks) which could be argued to be complete and representative of the full breadth/scope of ethical challenges posed by XR.

 Regarding the inclusion criteria, I'm a bit uncertain about where the line is drawn between a source identifying itself as an ethics guideline that would inform the review. I ask because some of our groups work treads close to this line e.g.

- Augmenting People, Places & Media: The Societal Harms Posed by Everyday Augmented Reality, and the Case for Perceptual Human Rights | Request PDF (researchgate.net)
- (PDF) Big Buddy: Exploring Child Reactions and Parental Perceptions towards a Simulated Embodied Moderating System for Social Virtual Reality (researchgate.net)
- (PDF) The Dark Side of Perceptual Manipulations in Virtual Reality (researchgate.net)
- The Dark Side of Perceptual Manipulations in Virtual Reality | Proceedings of the 2022 CHI Conference on Human Factors in Computing Systems (acm.org)
- ohagan2021ismar.pdf (mkhamis.com)

I don't say this as a typical "my papers should be included" reviewer comment, but rather that I think the inclusion criteria needs to be a bit clearer and more precise here to make clear why such papers were excluded, and what this means for how comprehensive the findings are. I think what the inclusion criteria (Table 2) are really arguing for is only examining papers that prescribe a general ethics framework or series of guidelines, rather than examining papers that examine and provide guidelines only on specific ethical risks with a narrow scope (e.g. mediated perception, child safety, perceptual manipulation). Moreover, this also precludes quite comprehensive review papers such as: 

-  
Security and Privacy Approaches in Mixed Reality:A Literature Survey (arxiv.org)

This, then, does provide an obvious limitation that should be highlighted more strongly - the breadth and depth of the fundamental values and dominant principles identified in the review is really contingent on there being up-to-date assessments of the ethical risks posed, as considered across disciplines, and there is a natural (and significant) lag between research that identifies specific (and perhaps more niche) ethical challenges (particularly prevalent in HCI where research is quite rapidly pushing the boundaries here), and the incorporation of those challenges into general frameworks. For example, I doubt any framework hugely addresses the risks, harms, and recommendations around *memory manipulation* as covered by e.g. Elise Bonnail; *dark patterns* as covered by Veronika Krauß; or *child safety* as covered by e.g. Cristina Fiani and Divine Maloney, to name but a few.

 So in the summary of ethical issues here, there are significant gaps/blindspots - and the risk is that the reader sees this scoping review as a summation of all ethical risks across the literature that have so far been identified, which isn't the case. The "gaps in the literature" and "limitations" sections don't really address this sufficiently for me. And indeed there should be consideration of how we as a community should move towards a more complete understanding of the rapidly emerging ethical risks here. The "recommendations for moving forward" do somewhat contribute here, but *how* will we go about making a comprehensive framework given the breadth of risks/harms posed? That is the big challenge that should be addressed more strongly. 

 Finally, the recommendations the paper ends on are rather generic and not well motivated, really espousing best practice in research generally, rather than specific to ethical challenges provoked uniquely by XR e.g. protection of research participants is paramount in any ethical research - and what about protection of XR users? I'd suggest removing the recommendations really, and concentrating on the bigger picture: how we establish and agree more comprehensive and agile ethical frameworks for XR, and why such a framework is necessary.

Is the study design appropriate and does the work have academic merit?

Yes

Is the work clearly and accurately presented and does it cite the current literature?

Partly

If applicable, is the statistical analysis and its interpretation appropriate?

Not applicable

Are all the source data underlying the results available to ensure full reproducibility?

Yes

Are the conclusions drawn adequately supported by the results?

Partly

Are sufficient details of methods and analysis provided to allow replication by others?

Yes

Reviewer Expertise:

Human-Computer Interaction and Extended Reality.

I confirm that I have read this submission and believe that I have an appropriate level of expertise to confirm that it is of an acceptable scientific standard, however I have significant reservations, as outlined above.

# Response

**Published:** 2025-02-07

**Authors:** Shereen Cox

**Affiliations:** Universitetet i Oslo, Oslo, Oslo, Norway

**The reviewer is correct in that this review was limited to papers that prescribe ethics frameworks or guidelines, codes etc and did not include papers that broadly examine general ethical risks.**

**The reason for this is the organisation of the project’s work package where one task objective was focused on existing ethics guidelines while another task objective sought to examine risks and harms generally. For this reason, papers mentioned by reviewer were in fact included in the second task and not for this one. This was made clear in the limitations.**

**Additionally, we acknowledge that the recommendations presented were general and not specifically tailored to unique ethical issues within XR. However, these recommendations were derived from the reviewed documents, not proposed by the authors.**

**We have revised the relevant section to clarify this and address any misunderstanding regarding the source of the recommendations.**
